# The role of vision and proprioception in self-motion encoding: An immersive virtual reality study

**DOI:** 10.3758/s13414-021-02344-8

**Published:** 2021-08-02

**Authors:** Rena Bayramova, Irene Valori, Phoebe E. McKenna-Plumley, Claudio Zandonella Callegher, Teresa Farroni

**Affiliations:** 1 Department of General Psychology, University of Padova, Via Venezia 8, Padova, Italy; 2 Department of Developmental Psychology and Socialization, University of Padova, Via Venezia 8, Padova, Italy; 3 School of Psychology, Queen’s University Belfast, Belfast, BT9 5BN UK

**Keywords:** Proprioception, Multisensory integration, Memory, Immersive virtual reality, Spatial cognition

## Abstract

Past research on the advantages of multisensory input for remembering spatial information has mainly focused on memory for objects or surrounding environments. Less is known about the role of cue combination in memory for own body location in space. In a previous study, we investigated participants’ accuracy in reproducing a rotation angle in a self-rotation task. Here, we focus on the memory aspect of the task. Participants had to rotate themselves back to a specified starting position in three different sensory conditions: a blind condition, a condition with disrupted proprioception, and a condition where both vision and proprioception were reliably available. To investigate the difference between encoding and storage phases of remembering proprioceptive information, rotation amplitude and recall delay were manipulated. The task was completed in a real testing room and in immersive virtual reality (IVR) simulations of the same environment. We found that proprioceptive accuracy is lower when vision is not available and that performance is generally less accurate in IVR. In reality conditions, the degree of rotation affected accuracy only in the blind condition, whereas in IVR, it caused more errors in both the blind condition and to a lesser degree when proprioception was disrupted. These results indicate an improvement in encoding own body location when vision and proprioception are optimally integrated. No reliable effect of delay was found.

**Electronic supplementary material:**

The online version of this article (10.3758/s13414-021-02344-8) contains supplementary material, which is available to authorized users.

## Introduction

Living in complex, large-scale environments makes it vital for us to continuously update our knowledge about our body’s location in space relative to various frames of reference. This involves processing and integrating information from different sensory modalities, including proprioception (the sense of the position and movement of our body in space) and vision. Inputs from these senses, be they together or separate in unisensory settings, need to be optimally encoded to form internal representations that can be used to guide spatial awareness and navigation (Klatzky, 1980). The contributions of vision and proprioception to retrieving information about own body location and movement at different stages of remembering, i.e., encoding, storage, and recall phases, are unknown. Understanding these complex relationships is crucial for the development of technologies that facilitate human navigation and to inform motor rehabilitation programs.

### Combination of vision and proprioception and proprioceptive in memory for self-motion

One question that remains to be answered is whether a combination of visual and proprioceptive cues aids in recall in self-motion (note that we refer to self-motion to imply one’s own body movement, regardless of whether it was self- or other-induced). It is not difficult to imagine that we generally utilize both senses for successful motor activity, given the common anecdotal experience of stumbling and hitting into objects when navigating without vision, such as when attempting to walk around in dark environments or with disrupted proprioception, for example in cases of alcohol intoxication (Modig, 2013). This advantage of multisensory integration is supported by previous research demonstrating that estimations of angular displacement are less variable when participants are provided with richer cross-modal sensory input (Bakker, Werkhoven, & Passenier, 1999; Jürgens & Becker, 2006). By investigating participants’ judgements of trajectory geometry, Reuschel et al., (2010) showed that perception is more precise in multisensory visuo-proprioceptive conditions compared to unisensory visual and proprioceptive ones. Although there have been examples showing that optic flow alone can be sufficient to complete a return-to-origin task, the introduction of body-based cues (proprioceptive and vestibular) when walking through the virtual environment led to decreased variability in responding (Kearns et al., 2002). Interestingly, this improved precision did not depend on the amount of optic flow, which suggests a stronger reliance on proprioceptive cues. Studies focusing on purely rotational movements and that compared unisensory visual (only optic flow available, no movement) and proprioceptive conditions indicate that proprioceptive information appears to be quite important (Bakker et al., 1999; Lathrop and Kaiser, 2002; Riecke, Cunningham, & Bülthoff, 2007). Similar findings hold for purely linear movements and more complex motor operations (for a review, see Campos & Bülthoff 2012).

Importantly, Valori et al., (2020) found that accuracy in visuo-proprioceptive conditions was much higher than in conditions providing only proprioception and slightly higher than in conditions where only vision was available. The authors focused on vision and proprioception as the main senses providing information for whole-body displacement in space. Participants were passively rotated and then actively rotated themselves back to the point where the passive rotation started (Valori et al., 2020). In this case, although vestibular information from semicircular canals about head motion plays a big role, it needs to be combined with neck proprioception to signal the trunk’s position and motion relative to the head. Moreover, semicircular canals are never stimulated alone, which is why vestibular information can be considered not the sole most reliable cue but rather a building block of the wider combination of sensory cues forming the somatosensory perception of self-motion (Israel and Warren, 2005).

Notably, benefits of multisensory input for short-term memory have been previously demonstrated for different combinations of sensory inputs. For example, Fougnie and Marois (2011) found that memory for audiovisual stimuli was better than for unimodal visual or auditory stimuli. They interpreted these results as evidence of separate memory stores for each sensory modality that additively contributed to forming a multisensory object in memory, whereas other authors explain such findings in terms of the pure benefit of multisensory cue combination in working memory (e.g., Delogu et al., 2009; Quak et al., 2015). Given the lack of research into the role of visuo-proprioceptive cue combination in working memory, there is scope to explore whether there is an advantage of multisensory input in proprioception-based memory tasks. When it comes to comparing performance in unisensory conditions, one of the reasons for higher accuracy with one sensory modality compared to the other could be more efficient encoding or easier storage of stimuli (for example, through the formation of richer representations) in that specific modality.

### Movement amplitude versus recall delay in proprioceptive tasks

As mentioned earlier, another question is which stage of remembering is most affected in the reproduction of whole- body angular displacement. As in other memory-based tasks, proprioceptive tasks involve three stages of remembering: encoding, storage, and recall (Shiffrin & Atkinson, 1969). For example, in a self-turning task, encoding means capturing the sensory information and it is affected by movement amplitude, which represents the amount of information to be encoded, whereas storage would refer to maintaining this information online for manipulation and/or recall. It is affected by recall delay, which is the amount of time that passes from encoding to recall. Previous research has illustrated that the effect of encoding can be differentiated from the storage phase in studies that aim to explore aspects of proprioceptive memory (Klatzky et al., 1990; Lemay & Proteau, 2001; Loomis et al., 1993). To investigate the encoding phase, most studies on spatial memory have focused on memory for external objects in relation to the body, mainly involving reaching and grasping movements. For example, (Lemay & Proteau, 2001) showed that movement amplitude has a bigger effect on the accuracy of manual aiming to remembered targets than recall delay. Several other studies support this finding, reporting that spatial error (Adam et al., 1993; Adamovich et al., 1998) and sometimes spatial variability (Lemay & Proteau, 2002; Messier & Kalaska, 1997; Prablanc, Pelisson, & Goodale, 1986) of reaching movements to remembered targets increases with increasing target distance. It is, however, not clear whether the same distance effect would be observed for movement amplitude in reproducing whole-body angular displacements without a visual target reference. Previous findings (Valori et al., 2020) seem to partially support the hypothesis of the role of encoding, although the interaction with sensory and environment conditions was not explored.

On the other hand, when the storage phase is manipulated without any experimental manipulation of the amount of the information to be encoded, research on aiming arm movements has shown that accuracy in recalling visuospatial information can decay rapidly (Elliott & Madalena, 1986; Hesse & Franz, 2009; Hu, Eagleson, & Goodale, 1999). Lemay and Proteau (2002) have shown that even a recall delay of 2 s can lead to significantly higher error rates. As for the memory for target-directed locomotion, although Thomson (1983) found that spatial short-term memory can be highly accurate up to 8 s in a series of experiments, later studies failed to replicate these results (Elliott, 1986; Elliott and Madalena, 1986; Steenhuis & Goodale, 1988). In fact, in the study conducted by Steenhuis and Goodale (1988), it was the distance participants had to walk to the target that affected short-term memory. Furthermore, in studying vestibular memory, Israël et al., (1991) found that performance only starts to decline at delays of 5 min.

One seminal work on spatial memory that could potentially explain the results regarding the amount and type of information to be encoded in short-term memory is the so-called “encoding-error model” for non-visually guided pathway completion by Fujita et al., (1993). It postulates that the error in producing a target-directed locomotion arises from the imprecise internal representations formed by the participant, rather than execution. The model is supported by findings of near-perfect performance in studies where participants were first presented with the target and then walked to it without vision (Elliott and Madalena, 1986; Loomis, Da Silva, Fujita, & Fukusima, 1992; Rieser, Ashmead, Talor, & Youngquist, 1990). Fujita et al., (1993) argue that since vision is available for optimally encoding the pathway, errors should be expected to occur almost exclusively at the execution stage. This model, however, is based on non-visual pathway completion and it is not clear whether encoding relies more heavily on vision or proprioception (while vestibular information is consistent across conditions) or if both senses contribute equally. Shedding light on this would be crucial given that, in our daily life, we execute many tasks beyond blindly walking towards visually encoded objects.

### IVR: An altered sensory experience

Immersive virtual reality is frequently provided through a wearable head-mounted display (HMD). The main advantage of using IVR is that it allows for a broad range of manipulations of information coming from individual sensory systems, be they visual, vestibular, or proprioceptive, all of which are usually bound together in spatial cognition and navigation. This makes it possible to study the contribution of these individual sensory inputs and of multisensory integration to self-perception and motor control (Campos & Bülthoff 2012; Mohler, Campos, Weyel, & Bülthoff, 2007; Sanchez-Vives & Slater, 2005). A number of sensory conflicts can be created by means of IVR technologies, since users’ expectations about the outcomes of their actions, which are based on what they see in the virtual environment, are not always met given the boundaries of the real world around them. People move and orient themselves differently in IVR compared to reality (Mohler et al., 2007; Riecke & Wiener, 2007; Valori et al., 2020) and it has been suggested that IVR may bring about a sensory conflict between proprioception and vision which affects users differently depending on their reliance on one of the two modalities (Prothero & Parker, 2003). Visuo-proprioceptive conflict can affect different aspects of motor functioning. For example, Chiarovano et al., (2015) studied postural stability in IVR and found that proprioception can be distorted via the distracting moving dots to such a degree that participants could not maintain their balance on a Wii Balance Board. This and similar findings point to the possibility of inducing proprioceptive challenges through the manipulation of vision. Such a possibility would greatly facilitate and accelerate research on self-motion in environments with distorted proprioception given that directly manipulating proprioceptive cues is usually more challenging logistically.

Recently, several studies have examined whether memory in IVR is similar or dissimilar from Reality (Krokos, Plaisant, & Varshney, 2019; Kisker, Gruber, & Schöne, 2019). Although it has been found that episodic memory can be enhanced in IVR with the help of virtual mind palaces and by reproducing the episodic context (Krokos et al., 2019, 2019), less is known about memory for self-motion. The growing research and commercial use of this technology. To this end, the current study aimed to shed light on the role of memory in self-motion and how it might be compared to the same motion performed in reality.

### The present study

The present exploratory research aims to disentangle several potential aspects of memory for own body location. The main question of the present study was whether integration of vision and proprioception is beneficial for encoding the movement (i.e., the rotation) and if an advantage of this integration is also evident during the storage phase. Participants had to rotate themselves back to a specified starting position in three different sensory conditions: a blind condition, a condition with disrupted proprioception, and a condition where both vision and proprioception were reliably available. To answer the first part of the question, we tested whether larger rotations selectively impair performance in any of our sensory conditions. Given the higher error rate in both blind conditions (reality and IVR) in Valori et al., (2020), it was hypothesized that increasing amplitude would affect this condition more than others. As regards the difference in error rates between the conditions where vision and proprioception are both available versus the unisensory-like visual condition where proprioception is disrupted, two scenarios could be expected. First, if our disruption of proprioception resulted in a decline in accuracy, then it might also be more challenging to encode larger rotations in that case than in the visuo-proprioceptive condition. No difference between these conditions was to be expected if the manipulation did not affect performance, as this condition would then resemble the multisensory setting. However, given mixed findings on the role of vision and proprioception in different aspects of self-motion and reaching movements (Bakker et al., 1999; Jürgens and Becker, 2006; Sarlegna & Sainburg, 2009; Sober & Sabes, 2003; Valori et al., 2020), one possibility remains that vision is dominant for self-motion estimation. In this case, performance in the visual condition could be expected to be similar to that in the condition where vision and proprioception can be optimally integrated.

As findings on the manipulation of the proprioceptive storage phase, such as the effect of recall delay on proprioceptive accuracy, are mixed and far from conclusive, we decided to focus on delays of small durations to investigate whether short-term retention of start position would benefit from sensory memory contributions. Three delay durations were introduced into the storage phase: no delay, 3-s delay, and 6-s delay. If there are benefits of cue combination during the storage phase, we would expect longer delays to affect performance more than shorter delays to a higher degree in unisensory versus multisensory conditions.

In this study, IVR is used as a potential tool with which to manipulate proprioception and vision and compare this condition with a multisensory condition and disentangle the influence of each modality on memory for own body orientation. One possibility is that IVR could be more effective at creating an environment with proprioceptively uninformative visual cues (e.g., a spherical room) such that participants could be proprioceptively disoriented without direct manipulation of somatosensory cues. Furthermore, we aimed to shed light on the role of memory in self-motion performed either in IVR or in reality.

## Materials and methods

### Participants

Forty-eight adults (35 females) took part in this experiment. The mean age of participants was 21.59 years (SD = 0.35, range = 18–26) and 40 participants were undergraduate or postgraduate psychology students. The inclusion criteria were not having any history of neurological disease or other conditions affecting cognitive functioning, and being under 40 years of age. Older participants were excluded due to the known decay of short-term memory capacity, but also based on the literature that suggests a downward trend in proprioceptive ability with advancing age (Hurley et al., 1998, 40-60 years of age;Wingert et al., 2014).

### Apparatus and conditions

**Fig. 1:**
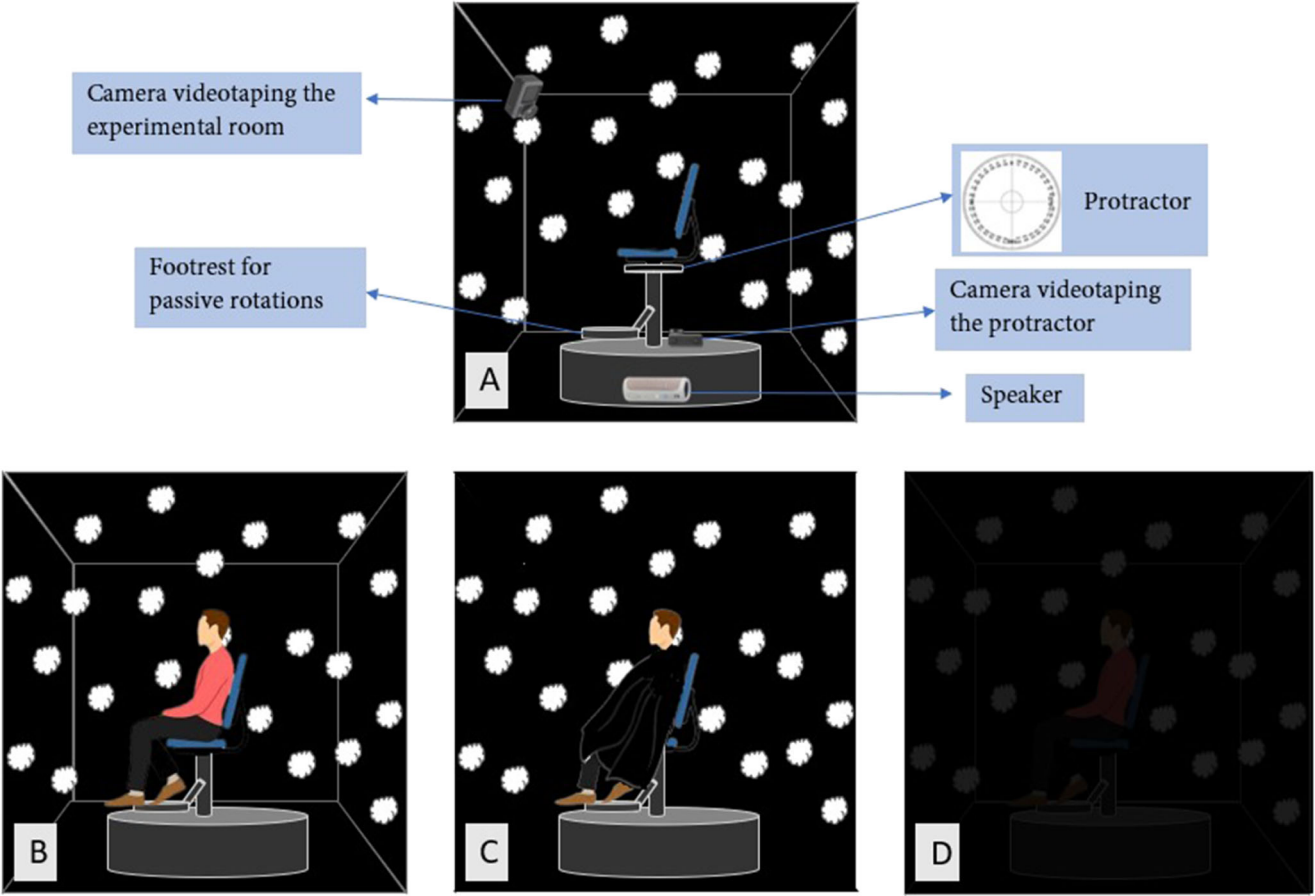
**A** Experimental room, interior. The swivel chair is in the center of the room with a protractor and a camera videotaping the protractor located under it. **A** R_VP: the swivel chair in the visuo-proprioceptive real environment; **B** R_V: a participant wearing the black poncho in the ‘vision’ real environment. UV light on; **C** R_P: a participant in complete darkness in the ‘proprioception only’ condition. A Nikon KeyMission 360 camera was used to create 360^∘^ images of the room and to build the IVR. Therefore, participants saw the same environment in the IVR conditions

A testing room designed to control the visual landmarks available during the task was used. Inside the room, there was a swivel chair that was fixed to the floor on a round platform (Fig. 1). A 360^∘^ protractor under the seat was visible via a dedicated camera which allowed the measurement of the degree of each rotation. For one of the conditions (see condition R_V in the description of conditions below), a UV lamp (E27 26W) was used to obscure other visual stimuli such that the white clouds on the walls were the only visual cues available. With the UV light on, participants were asked to wear a black poncho that covered their bodies, depriving them of the first-person view of their body (Fig. 1). The IVR simulation was provided through an HMD. We used Oculus Gear VR 2016, 101^∘^ FOV, 345 g weight, interfaced with a Samsung Galaxy S7 (ANDROID 8.0.0 operating system). For a full description of the technical apparatus and experimental setup, see Valori et al., (2020).

Participants performed a self-turning task in a 3 (Perception: vision, proprioception, vision+proprioception) X 2 (Environment: reality vs. IVR) X 2 (Amplitude: 90-degree rotations vs 180-degree rotations) X 3 (Delay: no delay, 3-s delay, 6-s delay) within-subjects design. The same three sensory conditions were completed in a real environment (R) and in IVR. In each of these two environments, one blind condition removed all visual information such that only proprioceptive information could be used (P condition). Another condition limited the access to visual landmarks (removing visual information about the body and corners of the room while retaining the overall use of vision) in order to disrupt proprioception. This condition was called V (i.e., vision) for the sake of convenience, and it should be noted that although proprioception was disrupted, the proprioceptive input from both passive and active rotations was still present. The experimental manipulation in this condition consisted of removing participants’ view of the room’s corners and of their own body such that they would not be available as visual cues to inform proprioception. Finally, there was a condition where reliable visual and proprioceptive information were available (VP condition), which in our case consisted of the brightly lit experimental room. Participants performed blocks of six trials in the perceptual-environmental conditions depicted in Fig. 1.

We aimed to control whether rotation amplitude and recall delay would affect performance. For this purpose, each condition was run six times: with two angle amplitudes (approximately 90 and 180 degrees) and with three delays (0, 3, and 6 s), totaling 36 trials (each constituting an independent condition) per participant. As the passive rotation was manually performed by the experimenter, perfect accuracy in reaching 90 and 180 degrees was not possible. Given the variability in the actual passive rotations, we considered amplitude as a continuous variable. While the passive rotations were performed manually, all experimenters were trained to perform consistently. Correlation analysis indicated that the duration of passive turns and their amplitude were highly correlated (*r*(1721) = 0.62,*p* < .001), showing good consistency across trials. Furthermore, the passive rotation was made in both directions (clockwise and counterclockwise). Note that since, in our previous study (Valori et al., 2020), we found that direction has no effect on performance, direction was randomized and coupled with delay but not with amplitude, which means that it did not affect the total number of trials. The order of the six perceptual-environmental blocks described above was randomized.

The order of conditions was randomized. Participants performed blocks of six trials per condition. We aimed to control whether the rotation direction and amplitude would affect performance. For this purpose, each condition was performed twice: the passive rotation was made in both directions (clockwise and counterclockwise), and with two angle amplitudes (90 and 180 degrees). As the passive rotation was manually performed by the experimenter, perfect accuracy in reaching 90 and 180 degrees was not possible. Given the variability in the actual passive rotations, we considered amplitude as a continuous variable. Furthermore, each condition was run with three different delays: 0, 3, and 6 s. Participants completed the task with both rotation amplitudes in each delay condition, totaling to six rotations per Environment and Perception condition. We counterbalanced the amplitudes order between subjects. We tested the ABAB sequence in 50% of subjects and the BABA sequence in 50% of subjects.

Note that the rotation amplitudes were kept at 90 and 180 degrees as in our previous study (Valori et al., 2020) given that the delay durations here are relatively short and larger amplitude might require longer delays (Lemay and Proteau, 2002).

### Measures of task performance

The accuracy of self-turn performances was calculated in terms of error as the absolute difference between the start position (from which the experimenter started the passive rotation) and the return position (in which the participant stopped the active rotation). Accuracy was manually measured during an offline coding of the video recording. Two independent evaluators coded the videos and entered the start and return positions in the dataset. Values which were divergent for more than two degrees were a priori considered disagreement values. A third coder examined the video records of the disagreement values to make the final decision. In case of a disagreement value, the third coder’s value replaced the value that differed most from the third coder’s value. We obtained a dataset with two codes for each datapoint. The intercoder agreement was calculated by conducting an intra-class correlation (ICC), which estimated a nearly perfect agreement of 0.99.

### Procedure

Participants were welcomed to the lab and asked to sign a consent form. Afterwards, participants were invited into the testing room and asked to sit on the swivel chair which was fixed in the middle of the recording area inside the room. Two experimenters were present to run the experiment. The first experimenter would stay inside the testing room to control the passive rotation and remain silent behind the participant, providing no visual or auditory cues. The second experimenter managed the experiment from outside the testing room by changing the conditions and controlling the video recording of the experiment. The second experimenter also monitored the video feed and gave verbal instructions to the first experimenter and to the participants.

The experiment involved a self-turn paradigm in which the experimenter rotated the chair a certain degree (passive rotation) from a start position to an end position. After each passive rotation, a beep sound cued participants to rotate back to the start position (active rotation). This cue played at three delay durations: immediately, after 3 s, or after 6 s. During the passive rotation, participants kept their feet on a footrest which rotated along with the chair. In this way, they could not make steps while being rotated, and could not simply count the number of steps to make active rotations. To perform the active rotations, participants could use their feet on the still platform under the chair to move themselves. We did not manipulate vestibular information, which remained consistent across all experimental conditions.

### Statistical approach

To evaluate the potential ways in which memory issues could influence proprioceptive accuracy, two sets of analyses were conducted using a Bayesian model selection approach (McElreath, 2016).

In the analysis, we considered the influence of amplitude, delay, and sensory and environmental conditions on the accuracy of self-turn performance. This allowed us to investigate the research goal: whether integration of vision and proprioception is crucial only for encoding the movement or also for the storage of the movement.

Starting from a full model that included all the interactions and variables of interest, predictors were removed until the most plausible model was obtained according to information criteria (Wagenmakers & Farrell, 2004; Yamashita, Yamashita, & Kamimura, 2007). The Watanabe-Akaike Information Criterion (WAIC, Gelman et al., 2014; Vehtari et al., 2017) was used as information criteria to evaluate the models. WAIC is the corresponding Bayesian version of the commonly used Akaike information criterion (AIC, Akaike 1973) and lower WAIC values indicate a better model. The selected models were interpreted by means of estimated parameters, graphical representations, and planned comparisons.

Bayesian generalized mixed-effects models were used as they allow for the complex structure of this data (Gelman et al., 2013). Specifically, data are characterized by: (1) a continuous non-normally distributed dependent variable (i.e., self-turn error); (2) within-subject factors (i.e., Perception and Environment conditions); (3) a quantitative independent variable (i.e. Amplitude of the passive rotation); (4) and a within-subject factor (i.e., Delay). Random intercepts were included to account for participants’ interpersonal variability, while the other variables were considered as fixed effects. Gamma distribution, with logarithmic link function, was specified as the family distribution of the generalized mixed-models to account for the distribution of the data.

Analyses were performed using the R software version 3.6.1 (Team & et al. 2018). Models were estimated using the R package *brms* (Bürkner & et al. 2017). All models used default prior specification of the R package brms. Detailed prior specifications are reported in the supplemental online material.

## Results

The detailed report of the results of the analysis is presented in the Supplemental Material (SM).

### Descriptive statistics

The dataset included start, end, and return positions based on which the angle of passive rotations and accuracy of active rotations were calculated. The intra-class correlation index (ICC) was calculated to evaluate the inter-coder reliability. ICC estimates and their 95% confidence intervals (CI) were calculated based on a mean-rating ($\text {k} {}={} 2$), consistency, two-way mixed-effects model. The analysis estimated an $\text {icc} {\kern -3.5pt}={\kern -3.5pt} .99$ (95%CI 0.99–0.99). This high reliability was obtained thanks to the low mean difference between coders’ values (0.28) within the wide range of possible values (0/360). $\text {icc} {}={} .99$. This nearly perfect inter-coder agreement derives from the small mean difference between the two coders’ values, within the huge range of possible values (0/360).

Out of the 48 participants, 41 participants completed the task in all 36 trials, and five participants completed 35 trials due to technical issues. Thus, the final data consists of 1723 observations nested in 48 participants.

**Fig. 2:**
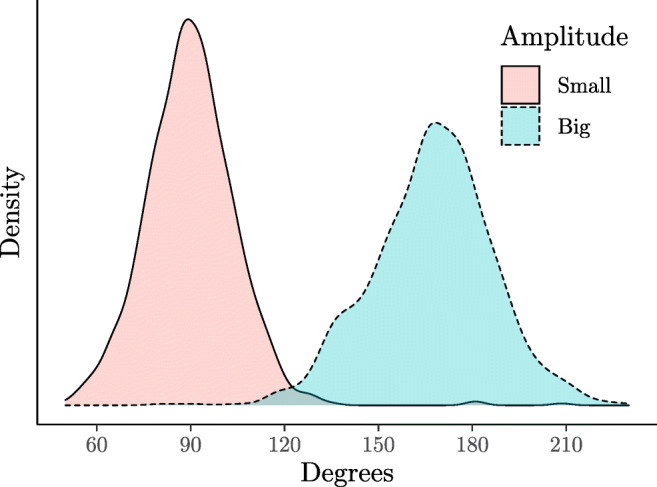
Amplitude distribution of the actual passive rotations (i.e., task difficulty; *n*_*participants*_ = 48; *n*_*observations*_ = 1723)

Amplitude was considered as a continuous variable (Fig. 2). To facilitate the interpretation of the results, this variable was standardized. Considering amplitude as a continuous variable rather than a dichotomous categorical variable guarantees that results are not influenced by the variability in the actual rotation between the different experimental conditions. Although the distributions are slightly different, all of them cover appropriately the same range of values (see SM).

**Fig. 3:**
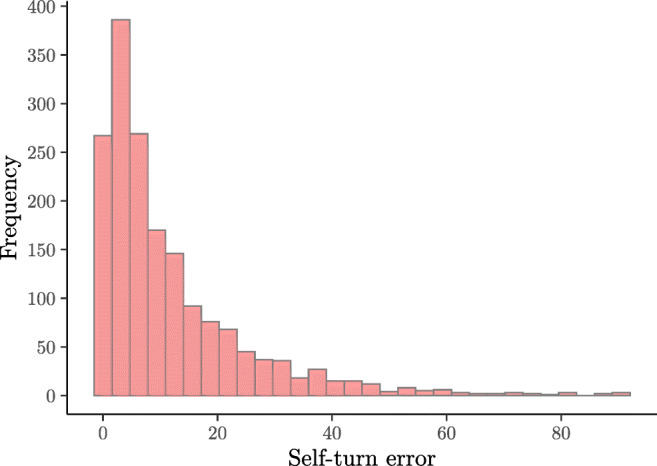
Frequencies of the observed self-turn errors (*n*_*participants*_ = 48; *n*_*observations*_ = 1723)

The frequency of observed self-turn error values is reported in Fig. 3.

The means and standard deviations of self-turn error for the six experimental conditions with different delays are reported in Table 1. For the sake of interpretability, descriptive statistics were marginalized over the variables Amplitude and Direction, and Amplitude is explored later on in the analysis.

**Table 1:** Descriptive statistics. Means and standard deviations of self-turn error according to experimental conditions

	Proprioception		Vision		Vision + Proprioception		Total	
	Mean	SD	Mean	SD	Mean	SD	Mean	SD
Reality								
0 s	16.6	9.8	6.3	5.2	5.0	4.1	9.3	4.0
3 s	17.5	12.1	5.0	3.9	5.4	3.4	9.5	5.6
6 s	17.4	11.1	5.4	3.4	5.8	5.5	9.5	4.6
Total	17.2	7.3	5.6	2.9	5.4	3.1	9.4	3.0
IVR								
0 s	19.1	10.4	14.7	7.8	7.4	5.3	13.7	4.5
3 s	17.8	9.9	15.1	9.7	11.4	9.6	14.8	6.1
6 s	20.8	12.3	13.0	8.5	9.5	8.3	14.5	6.1
Total	19.1	8.1	14.3	6.6	9.4	5.9	14.3	4.9
Total								
0 s	17.7	7.9	10.6	5.3	6.1	3.5	11.5	3.2
3 s	18.2	10.8	10.1	5.3	8.5	5.2	12.1	4.8
6 s	19.0	8.6	9.4	4.8	7.7	5.1	12.0	3.9
Total	18.3	6.5	10.0	3.8	7.4	3.4	11.9	3.3

*Note:* IVR = immersive virtual reality. *n*_*participants*_ = 48; *n*_*observations*_ = 1723

With regard to the marginal effect of Environment, participants were on average more accurate in the Reality conditions (m = 9.4, sd = 3.0) than in the IVR conditions (m = 14.3, sd = 4.9). With respect to the marginal effect of Perception, a difference in performance was found in one of the conditions depending on the Environment. In Reality, participants were more accurate when they could rely on both Vision+Proprioception (m = 5.4, sd = 3.1) or Vision specifically (n = 5.6, sd = 2.9) than when they had to rely solely on Proprioception (m = 17.2, sd = 7.3). In IVR, accuracy was still lowest in the Proprioception-only condition (m = 19.1, sd = 8.1). The error rate was, however, different in the two visual conditions, whereby performance was notably better in the Vision+Proprioception condition (m = 9.4, sd = 5.9) compared to when participants’ proprioception was unreliable (Vision condition) (m = 14.3, sd = 6.6).

### Model selection

The initial model included the self-turn error as the dependent variable. The independent variables were all the interactions, up to four-way interactions, between Environment, Perception, Amplitude, and Delay. The direction of the rotation and the random intercepts for each participant were also included. The WAIC value of the initial model was 11505.

The most plausible model was m.9 (waic = 11477), with a probability of being the best of .64, superior to all other models (< .20; see Supplemental Material). As independent variables, the model included the interaction between Environment, Perception, and Amplitude and the interaction between Perception and Delay. The random intercepts for each participant were also included in the model.

### Model interpretation

To evaluate the model fit (i.e., model’s ability to explain the data) we used a Bayesian definition of R-squared (Gelman, Goodrich, Gabry, & Vehtari, 2019). The estimated value of Bayesian R-squared for the model m.9 is 0.35 (95% BCI = 0.30; 0.40), that is, the model explained 35% of the variance of the data. Posterior Predictive Check (PPC; i.e., model ability to replicate the observed data) is presented in Fig. 10 in the SM. Overall, the model shows a good fit to the observed data.

To assess the relative plausibility of the effects included in the model, the evidence ratios were computed for each interaction effect. The selected model is 83 times more likely to explain the results than the model without the three-way interaction (Environment, Perception, and Amplitude), and 48 times more likely than the model without the two-way interaction (Perception and Delay).

The 95% Bayesian credible intervals (BCIs) of the model parameters’ posterior distributions were evaluated. Ninety-five percent BCIs represent the range of the 95% most credible parameter values given the prior distribution and the observed data. Thus, an effect is considered to be present if the value zero is not included in the 95% BCI, whereas the presence of zero values in the 95% BCI is interpreted as no-effect. In order to interpret the effects of model m.9, the predicted mean values were computed for each combination of conditions and presented graphically.

### The interaction between rotation amplitude, Perception, and Environment

**Fig. 4:**
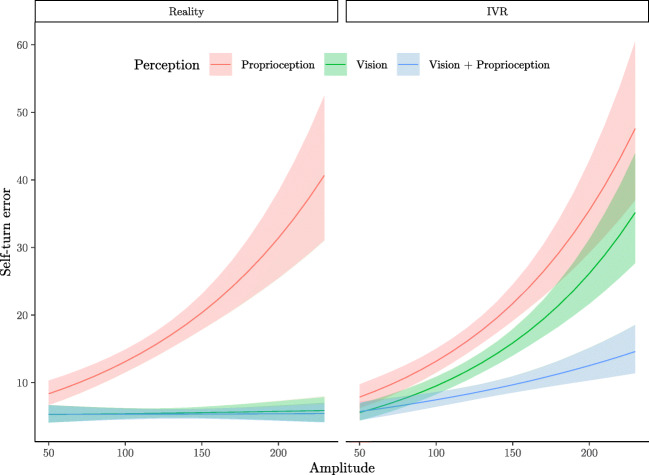
Predicted mean of self-turn error according to Amplitude in reality and in IVR (*n*_*participants*_ = 48; *n*_*observations*_ = 1723). The line represents the mean value, the shaded area the 95% BCI values

Self-turn error is differently influenced by Amplitude depending on Perception and Environment (see Fig. 4). In Reality, accuracy is expected to decline with the increasing of the Amplitude in Proprioception but not in the Vision and Vision+Proprioception conditions. In the IVR conditions, more errors are expected with increasing Amplitude in all Perception conditions.

Although Amplitude was considered as a continuous variable in the analysis, the values mostly clustered around 90 and 180 degrees. As such, the predicted mean values at 90 degrees and 180 degrees of rotation amplitude are reported in Table 2. We considered these rotation amplitude values as a reference, but predicted means can be computed for other values as well. To quantify the relative increase in the different conditions, the ratios of the self-turn errors between 180 degrees and 90 degrees of rotation amplitude are reported in Table 12 in SM.

**Table 2:** Predicted self-turn error mean according to experimental conditions

Conditions			Error	95 % BCI	
Environment	Amplitude	Perception	Mean	Lower	Upper
		Proprioception	11.99	10.42	13.79
		Vision	5.38	4.56	6.29
Reality	90	Vision + Proprioception	5.32	4.48	6.26
		Proprioception	26.40	22.25	31.19
		Vision	5.67	4.60	6.90
	180	Vision + Proprioception	5.37	4.01	6.35
		Proprioception	11.89	10.24	13.74
		Vision	8.57	7.35	9.93
IVR	90	Vision + Proprioception	7.08	6.01	8.29
		Proprioception	29.17	24.81	34.09
		Vision	21.44	18.33	24.93
	180	Vision + Proprioception	11.30	9.64	13.23

*Note:* IVR = immersive virtual reality. *n*_*participants*_ = 48; *n*_*observations*_ = 1723

### Recall delay

**Fig. 5:**
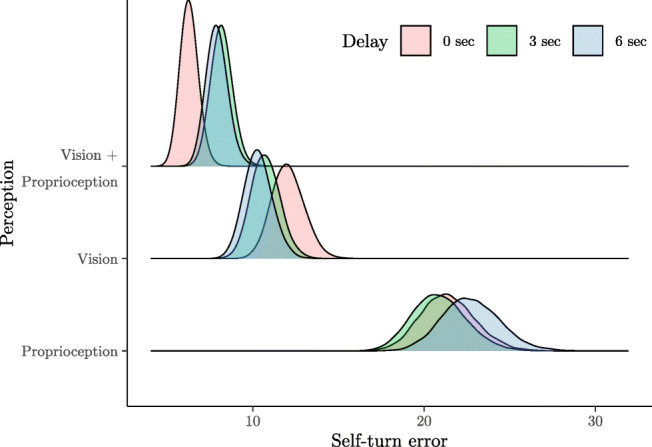
Distributions of the predicted means of self-turn error according to Perception and Delay (*n*_*participants*_ = 48; *n*_*observations*_ = 1723)

Considering the interaction between Perception and Delay the predicted mean values are presented in Fig. 5.

In the Proprioception and the Vision conditions, no differences are expected based on Delay. Only in Vision+Proprioception are more errors expected in the 3-s and 6-s Delay conditions compared to No delay. Predicted values are reported in Table 3. To quantify the relative difference between conditions, the ratios of the self-turn errors are reported in Table 15 in SM.

**Table 3:** Predicted self-turn error mean according to perception and delay conditions

Conditions		Error	95 % BCI	
Perception	Delay	Mean	Lower	Upper
Proprioception	0 s	21.39	18.45	24.66
	3 s	20.89	18.03	24.16
	6 s	22.75	19.61	26.24
Vision	0 s	12.06	10.34	13.97
	3 s	10.74	9.22	12.45
	6 s	10.31	8.81	11.98
Vision + Proprioception	0 s	6.28	5.39	7.30
	3 s	8.17	7.05	9.39
	6 s	7.90	6.81	9.15

*Note:* *n*_*participants*_ = 48; *n*_*observations*_ = 1723

## Discussion

The present study explored the role of visuo-proprioceptive integration in self-turn accuracy when compared to unisensory proprioceptive conditions and conditions with disrupted proprioception. It also compared the memory effects of movement amplitude and recall delay on proprioceptive accuracy in different sensory conditions and environments. In addition, this research added evidence for the possibility of disrupting proprioception through a manipulated IVR environment.

As expected, accuracy was lower in IVR in the sensory conditions where vision was available compared to the same conditions in reality. This is an important replication of previous findings (Valori et al., 2020) which suggests that results obtained from tasks completed in visual environments created and provided via IVR should be interpreted carefully with consideration of the fact that they may not mimic reality perfectly. One explanation for this drop in performance could be the difference in the way optic flow is updated in the two environments. In fact, it has been shown that the over- and underestimation of self-displacement can be compensated by manipulating optic flow field (Bruder et al., 2013). In addition, participants could not see their body in the IVR conditions as compared to the reality conditions where it is was in their field of view, which could lead to poorer performance in the visual conditions. Also in line with the results of Valori et al., (2020), the highest number of errors was observed in the blind condition in both environments. Similar to what has been shown in earlier studies on the role of vision in creating proprioceptively-guided movements (Lateiner and Sainburg, 2003; Sober & Sabes, 2003), these findings highlight the importance of visual information in guiding judgements about own body location and movement.

In contrast to the findings of Valori et al., (2020), where no difference was found between the two visual conditions, performance was worse in the IVR condition with disrupted proprioception than in the visuo-proprioceptive condition in IVR in the present study. One possible explanation for this inconsistency could be that the number of adult participants in the previous study was twice as small. This effect was not observed in the reality conditions that simulated this manipulation, where performance was similar to the multisensory condition. These results provide evidence that IVR can be used to disrupt proprioception by creating an environment where participants find it difficult to orient themselves, leading to increased errors when rotating back to a previously specified position. This effect could also be partially explained by the differences in optic flow, as mentioned above (Bruder et al., 2013). After removing the corners, which potentially served as the most prominent visual cues, participants had to focus on individual clouds that would enhance the use of optic flow. If the two environments differed in optic flow, removal of corners would cause a remarkable difference between the two conditions when other visual cues (more dependent on optic flow) would have to be used. In addition, although UV lights and a black poncho were used to remove participants’ view of their bodies in the V condition in reality, it is possible that such removal of the body from the field of view was not equal to the corresponding IVR condition where a view of the body was not present at all.

Furthermore, we found an interaction between sensory conditions, environmental conditions, and the effect of amplitude that was not investigated in the previous study. It is expected that 180-degree rotations lasted approximately twice as long as 90-degree rotations, which means that the encoding phase would have lasted longer. In reality, participants performed worse in reproducing larger rotation angles in blind conditions, whereas conditions where visual input was available were not affected by the degree of rotation. When it comes to the effect of larger rotation amplitude in IVR, there was almost no difference in the visuo-proprioceptive condition, a decrease in performance in the condition with disrupted proprioception, and an even higher decline in accuracy in the proprioception-only condition. These results suggest that when participants are provided with some visual landmarks, they can reliably use them for the estimation of angular displacement. Our finding that the encoding of angular displacement was more disrupted when vision was not available indicates that vestibular and proprioceptive information may not be as reliable as visual cues in longer travelled distances during self-rotation. This is in line with the encoding-error model of Fujita et al., (1993) for linear displacements which emphasizes the difficulty of creating an internal representation of the travelled path without vision during the encoding phase. With respect to the condition with disrupted proprioception, the interaction with amplitude provides evidence for the marked contribution of proprioception to optimal estimation of angular displacement. Taken together, these results point to an advantage of multisensory integration for encoding and thus recalling information about own location in space. In addition, although it has been suggested that error in angle reproduction could also be attributed to the execution phase (i.e., recall), only one study so far has provided evidence for such an effect in an attempt to disentangle encoding and execution errors and suggested that reproduction inaccuracies can largely be attributed to the execution phase (Chrastil and Warren, 2017). Moreover, counter to their expectations but in line with the current findings, in line with the current results, Chrastil and Warren (2017) found encoding error when participants had to reproduce 180-degree angles.

No clear trends were found with regard to different delay durations. Although we observed slightly higher accuracy in no-delay trials compared to 3- and 6-s delay trials in the visuo-proprioceptive condition, the actual difference in degrees is too small to represent a reliable advantage and improved performance. Taken together, results on recall delay seem to indicate that in the case of whole-body angular displacement, the advantage of multisensory input is evident only during the encoding phase and does not necessarily create more reliable memory traces which would improve storage. This finding may be task-specific, given that Lemay and Proteau (2002) found significantly higher error rates in memory for a target location after just 2 s. The lack of research on the effect of recall delay on estimating angular displacement did not allow for a good prediction of a delay duration that might decrease accuracy in our task. Therefore, our findings are useful in that they show that brief delays, up to at least 6 s, do not appear to influence recall of body position in adults. Earlier studies have shown that purely vestibular memory for the angular displacement of the head in remembering the position of external objects can be reliable up to one minute of recall delay, whereas small rotation angles (below 25 degrees) are overestimated after a delay of five minutes (Israël et al., 1991). Although our delays did not affect participants’ estimations of their body’s displacement, it is important to acknowledge that the delays were introduced into only one of the stages of remembering, namely storage, and that research which introduces delays at different stages may shed more light on this process. The duration at the start position may have varied slightly across participants, and these small differences in the length of the encoding phase may have influenced the results. However, the lack of association between delay duration and performance indicates that slight extension of the storage phase is not sufficient to affect performance. Research which introduces longer delays may shed more light on this process.

It is also important to mention that only two out of 48 participants reported slight nausea and dizziness. Since IVR-induced sickness (Saredakis et al., 2020) can affect results and thus seriously undermine research goals, this factor should always be considered in experimental design. In fact, we approached this point with a great degree of caution by minimizing the amount of time participants spent in the testing room as much as possible. However, given the feasibility of our paradigm in this respect, longer durations may be tested in future experiments.

Furthermore, although we cannot completely rule out that some participants might have used a counting strategy based on their time perception given that time and space are tightly linked in our perception of the world, we assume that to be unlikely in the present paradigm. Firstly, a passive rotation was used for encoding in order to avoid that participants count their steps and focus on this strategy. Secondly, such a strategy would not be optimally reliable due to the trial-to-trial changes in acceleration and speed of the experimenter-induced passive rotations and would be more efficient in experiments with more fixed duration to distance mapping.

One of the limitations of the present research was that the experimenter manually rotated the participant. Experimenters were trained to keep a consistent velocity and this velocity appeared not to vary dramatically. However, this was not completely consistent across trials and participants, potentially influencing participants’ performance to some degree as has been shown in previous research (Jürgens & Becker, 2006). Furthermore, aside from velocity, the time that elapsed during each passive rotation could have varied slightly, meaning that the length of the encoding phases was not constant. It is possible that small variations in this encoding period affected proprioceptive accuracy, although given the lack of effect of different delay durations, this is not expected to be the case.

These findings, apart from adding support to the findings that errors in reproducing whole-body displacements are attributable to the encoding process (Fujita, Klatzky, Loomis, & Golledge, 1993), are novel in that they suggest that accurate encoding is possible when visual and proprioceptive cues are combined. According to the encoding-error model and studies on multisensory integration (Bakker et al., 1999; Fujita et al., 1993; Jürgens & Becker, 2006; Reuschel et al., 2010), this could be explained by the more precise internal representation formed when information from different senses are combined to form a richer percept.

Future research might investigate whether a different trend in accuracy would be observed if participants actively performed both rotations themselves, which could indicate a benefit of richer somatosensory input during the encoding phase. Moreover, as mentioned previously, delays of longer durations could also be used in order to study the temporal limits of sensory information reliability in different sensory conditions. A possible caveat here would be whether deteriorated memory of body position after delays like, for instance, five minutes (Israël et al., 1991) or more would be informative for explaining cue combination or calibration in multisensory integration or spatial memory from a multisensory perspective.

## Conclusions

The present study indicates that precision in re-executing own body rotation remarkably decreases with larger rotations either when participants are deprived of vision or when their proprioception is manipulated to be unreliable. It was also found that the advantage of integrating information from vision and proprioception for subsequent recall of own body location is present during the encoding phase. Within 6 s, we report no effect of delay on storage. Overall, these results suggest that the integration of vision and proprioception aids encoding information about whole-body angular displacement. In general, as indicated in prior research, it is likely that the role of the perceptual weighting of the different sensory cues depends on the task demands and it might be true that in some tasks, cue combination is not that critical as in ours. Nevertheless, we assume that, in our daily life, the repertoire of self-motion tasks that rely solely on one sensory modality is limited. This research also shows that IVR can be used to disrupt proprioception. Such proprioceptive disorientation suggests exciting applications of IVR in sensorimotor research. For example, individuals with a heavier reliance on proprioception could be trained to use exteroceptive processing by making it more salient.

## Electronic supplementary material

Below is the link to the electronic supplementary material.

(PDF 1.76 MB)

## Data Availability

The data and supplemental materials for this experiment is available from the OSF repository at the following URL: https://osf.io/569x4/?view_only=1318de4be6fd4b378fb60f08de0d5281.

## Code Availability

The data analysis code for this experiment is available from the OSF repository at the following URL: https://osf.io/569x4/?view_only=1318de4be6fd4b378fb60f08de0d5281.

## References

[CR1] Adam JJ, Ketelaars M, Kingma H, Hoek T (1993). On the time course and accuracy of spatial localization: Basic data and a two-process model. Acta Psychologica.

[CR2] Adamovich SV, Berkinblit MB, Fookson O, Poizner H (1998). Pointing in 3D space to remembered targets. I. Kinesthetic versus visual target presentation. Journal of Neurophysiology.

[CR3] Akaike H (1973). Maximum likelihood identification of Gaussian autoregressive moving average models. Biometrika.

[CR4] Bakker NH, Werkhoven PJ, Passenier PO (1999). The effects of proprioceptive and visual feedback on geographical orientation in virtual environments. Presence: Teleoperators & Virtual Environments.

[CR5] Bruder, G., Wieland, P., Bolte, B., Lappe, M., & Steinicke, F (2013). Going with the flow: Modifying self-motion perception with computer-mediated optic flow. In *2013 IEEE International symposium on mixed and augmented reality (ISMAR)*. 10.1109/ISMAR.2013.6671765 (pp. 67–74): IEEE.

[CR6] Bürkner PC (2017). brms: An R package for Bayesian multilevel models using Stan. Journal of Statistical Software.

[CR7] Campos, J.L., & Bülthoff, H.H. (2012). Multimodal integration during self-motion in virtual reality. In *The neural bases of multisensory processes*. https://www.ncbi.nlm.nih.gov/books/NBK92853/: CRC Press/Taylor & Francis. 22593878

[CR8] Chiarovano E, De Waele C, MacDougall HG, Rogers SJ, Burgess AM, Curthoys IS (2015). Maintaining balance when looking at a virtual reality three-dimensional display of a field of moving dots or at a virtual reality scene. Frontiers in Neurology.

[CR9] Chrastil ER, Warren WH (2017). Rotational error in path integration: Encoding and execution errors in angle reproduction. Experimental Brain Research.

[CR10] Delogu F, Raffone A, Belardinelli MO (2009). Semantic encoding in working memory: is there a (multi) modality effect?. Memory.

[CR11] Elliott D (1986). Continuous visual information may be important after all: A failure to replicate Thomson (1983). Journal of Experimental Psychology: Human Perception and Performance.

[CR12] Elliott D, Madalena J (1986). The influence of premovement visual information on manual aiming. The Quarterly Journal of Experimental Psychology.

[CR13] Fougnie D, Marois R (2011). What limits working memory capacity? Evidence for modality-specific sources to the simultaneous storage of visual and auditory arrays. Journal of Experimental psychology: Learning, Memory, and Cognition.

[CR14] Fujita N, Klatzky RL, Loomis JM, Golledge RG (1993). The encoding-error model of pathway completion without vision. Geographical Analysis.

[CR15] Gelman, A., Carlin, J.B., Stern, H.S., Dunson, D.B., Vehtari, A., & Rubin, D.B. (2013). Bayesian data analysis. CRC Press. 10.1201/b16018

[CR16] Gelman A, Hwang J, Vehtari A (2014). Understanding predictive information criteria for Bayesian models. Statistics and Computing.

[CR17] Gelman A, Goodrich B, Gabry J, Vehtari A (2019). R-squared for Bayesian regression models. The American Statistician.

[CR18] Hesse C, Franz VH (2009). Memory mechanisms in grasping. Neuropsychologia.

[CR19] Hu Y, Eagleson R, Goodale M (1999). The effects of delay on the kinematics of grasping. Experimental Brain Research.

[CR20] Hurley MV, Rees J, Newham DJ (1998). Quadriceps function, proprioceptive acuity and functional performance in healthy young, middle-aged and elderly subjects. Age and Ageing.

[CR21] Israel, I., & Warren, W.H. (2005). Vestibular, proprioceptive, and visual influences on the perception of orientation and self-motion in humans. Head direction cells and the neural mechanisms of spatial orientation pp 347–381. http://www.cog.brown.edu/research/ven_lab/publications/IsraelWarren_Orientation_05.pdf

[CR22] Israël, I., Rivaud, S., Pierrot-Deseilligny, C., & Berthoz, A. (1991). “delayed vor”: An assessment of vestibular memory for self-motion. In *Tutorials in motor neuroscience* (pp. 599–607). Berlin: Springer, DOI 10.1007/978-94-011-3626-6_46.

[CR23] Jürgens R, Becker W (2006). Perception of angular displacement without landmarks: Evidence for Bayesian fusion of vestibular, optokinetic, podokinesthetic, and cognitive information. Experimental Brain Research.

[CR24] Kearns MJ, Warren WH, Duchon AP, Tarr MJ (2002). Path integration from optic flow and body senses in a homing task. Perception.

[CR25] Kisker, J., Gruber, T., & Schöne, B (2019). Experiences in virtual reality entail different processes of retrieval as opposed to conventional laboratory settings: A study on human memory. Current Psychology, pp. 1–8. 10.1007/s12144-019-00257-2

[CR26] Klatzky, R. (1980). Memory: structures and processes. San Francisco: WH Freeman & Co.

[CR27] Klatzky RL, Loomis JM, Golledge RG, Cicinelli JG, Doherty S, Pellegrino JW (1990). Acquisition of route and survey knowledge in the absence of vision. Journal of Motor Behavior.

[CR28] Krokos E, Plaisant C, Varshney A (2019). Virtual memory palaces: Immersion aids recall. Virtual Reality.

[CR29] Lateiner JE, Sainburg RL (2003). Differential contributions of vision and proprioception to movement accuracy. Experimental Brain Research.

[CR30] Lathrop WB, Kaiser MK (2002). Perceived orientation in physical and virtual environments: Changes in perceived orientation as a function of idiothetic information available. Presence: Teleoperators & Virtual Environments.

[CR31] Lemay M, Proteau L (2001). A distance effect in a manual aiming task to remembered targets: A test of three hypotheses. Experimental Brain Research.

[CR32] Lemay M, Proteau L (2002). Effects of target presentation time, recall delay, and aging on the accuracy of manual pointing to remembered targets. Journal of Motor Behavior.

[CR33] Loomis JM, Da Silva JA, Fujita N, Fukusima SS (1992). Visual space perception and visually directed action. Journal of Experimental Psychology: Human Perception and Performance.

[CR34] Loomis JM, Klatzky RL, Golledge RG, Cicinelli JG, Pellegrino JW, Fry PA (1993). Nonvisual navigation by blind and sighted: Assessment of path integration ability. Journal of Experimental Psychology: General.

[CR35] McElreath, R. (2016). Rethinking: an R package for fitting and manipulating Bayesian models. 10.1201/9781315372495

[CR36] Messier J, Kalaska J (1997). Differential effect of task conditions on errors of direction and extent of reaching movements. Experimental Brain Research.

[CR37] Modig, F. (2013). Effects of acute alcohol intoxication on human sensory orientation and postural control. Lund University.

[CR38] Mohler, B., Campos, J., Weyel, M., & Bülthoff, H.H. (2007). Gait parameters while walking in a head-mounted display virtual environment and the real world. In *13th Eurographics Symposium on Virtual Environments and 10th Immersive Projection Technology Workshop (IPT-EGVE 2007), (pp. 85–88). Eurographics Association*. http://hdl.handle.net/11858/00-001M-0000-0013-CCBB-0.

[CR39] Prablanc C, Pelisson D, Goodale M (1986). Visual control of reaching movements without vision of the limb. Experimental Brain Research.

[CR40] Prothero, J.D., & Parker, D.E. (2003). A unified approach to presence and motion sickness. In *Virtual and adaptive environments: Applications, implications, and human performance issues*, pp. 47. 10.1201/9781410608888

[CR41] Quak M, London RE, Talsma D (2015). A multisensory perspective of working memory. Frontiers in Human Neuroscience.

[CR42] Reuschel J, Drewing K, Henriques DY, Rösler F, Fiehler K (2010). Optimal integration of visual and proprioceptive movement information for the perception of trajectory geometry. Experimental Brain Research.

[CR43] Riecke, B.E., & Wiener, J.M. (2007). Can people not tell left from right in VR? Point-to-origin studies revealed qualitative errors in visual path integration. In *2007 IEEE Virtual Reality Conference*. 10.1109/VR.2007.352457 (pp. 3–10): IEEE.

[CR44] Riecke BE, Cunningham DW, Bülthoff HH (2007). Spatial updating in virtual reality: The sufficiency of visual information. Psychological Research.

[CR45] Rieser JJ, Ashmead DH, Talor CR, Youngquist GA (1990). Visual perception and the guidance of locomotion without vision to previously seen targets. Perception.

[CR46] Sanchez-Vives MV, Slater M (2005). From presence to consciousness through virtual reality. Nature Reviews Neuroscience.

[CR47] Saredakis D, Szpak A, Birckhead B, Keage HA, Rizzo A, Loetscher T (2020). Factors associated with virtual reality sickness in head-mounted displays: A systematic review and meta-analysis. Frontiers in human neuroscience.

[CR48] Sarlegna, F.R., & Sainburg, R.L. (2009). The roles of vision and proprioception in the planning of reaching movements. In *Progress in motor control*. 10.1007/978-0-387-77064-2_16 (pp. 317–335). Berlin: Springer. 10.1007/978-0-387-77064-2_16 PMC3709263 19227507

[CR49] Shiffrin RM, Atkinson RC (1969). Storage and retrieval processes in long-term memory. Psychological Review.

[CR50] Sober SJ, Sabes PN (2003). Multisensory integration during motor planning. Journal of Neuroscience.

[CR51] Steenhuis RE, Goodale MA (1988). The effects of time and distance on accuracy of target-directed locomotion: Does an accurate short-term memory for spatial location exist?. Journal of Motor Behavior.

[CR52] Team, R.C., et al. (2018). R: A language and environment for statistical computing. Vienna, Austria. https://www.R-project.org/

[CR53] Thomson JA (1983). Is continuous visual monitoring necessary in visually guided locomotion?. Journal of Experimental Psychology: Human Perception and Performance.

[CR54] Valori I, McKenna-Plumley PE, Bayramova R, Zandonella Callegher C, Altoè G, Farroni T (2020). Proprioceptive accuracy in immersive virtual reality: A developmental perspective. PloS One.

[CR55] Vehtari A, Gelman A, Gabry J (2017). Practical Bayesian model evaluation using leave-one-out cross-validation and WAIC. Statistics and Computing.

[CR56] Wagenmakers EJ, Farrell S (2004). AIC model selection using Akaike weights. Psychonomic Bulletin & Review.

[CR57] Wingert JR, Welder C, Foo P (2014). Age-related hip proprioception declines: Effects on postural sway and dynamic balance. Archives of Physical Medicine and Rehabilitation.

[CR58] Yamashita T, Yamashita K, Kamimura R (2007). A stepwise AIC method for variable selection in linear regression. Communications in Statistics—Theory and Methods.

