# Supplementary material for: The role of vision and proprioception in self-motion encoding: An immersive virtual reality study
Source: Atten Percept Psychophys. 2021 Aug 2;83(7):2865–78. doi: 10.3758/s13414-021-02344-8 (PMC8460581; doi:10.3758/s13414-021-02344-8)
Supplement: Supplementary file 1 — (PDF 1.76 MB) [file 13414_2021_2344_MOESM1_ESM.pdf]

# Integration of Vision and Proprioception Facilitates Encoding but not Storage: an Immersive Virtual Reality Study

Rena Bayramova[a], Irene Valori[b], Phoebe E. McKenna-Plumley[c], Claudio Zandonella Callegher[b], & Teresa Farroni[b]

University of Padova

[a] Department of General Psychology

[b] Department of Developmental Psychology and Socialization

[c] School of Psychology, Queen's University Belfast, Belfast, UK

**Submitted to *Psychological Research***

# Supplemental Materials

*Edited by*

Claudio Zandonella Callegher*

*e-mail address

claudiozandonella@gmail.com

Last compiled:

December 6, 2020

# Contents


**1 Introduction 1**

1.1 Study summary . . . 1

1.2 Supplemental material . . . 1

**2 Statistical Approach 2**

2.1 Bayesian model selection . . . 2

2.2 Bayesian generalized mixed-effects models . . . 3

2.3 Plan of the analysis . . . 4

**3 Data Presentation 5**

3.1 Delay as categorical variable . . . 6

**4 Descriptive Statistics 7**

4.1 Participants . . . 7

4.1.1 Participants' age . . . 7

4.2 Number of observations . . . 7

4.3 Amplitude . . . 8

4.3.1 Variability of the amplitude across conditions . . . 9

4.4 Passive duration . . . 10

4.5 Self-turn error . . . 11

4.6 Correlation beetween amplitude and duration . . . 14

**5 Model Selection 15**

5.1 Initial model . . . 15

5.1.1 Generalized Linear Model . . . 15

5.1.2 Model formula . . . 15

5.1.3 Models Priors . . . 16

5.1.4 Models Estimation . . . 18

5.2 Model selection steps . . . 18

**6 First Model Presentation 21**

6.1 Selected model . . . 21

6.1.1 Parameter posterior distributions . . . 21

6.2 Model Fit . . . 25

6.3 Effects evidence ratio . . . 26

6.4 Posterior effect distributions . . . 27

6.4.1 Interaction `Environment:Perception:Amplitude_st` . . . 27

6.4.2 Interaction `Perception:Delay` . . . . . . . . . . 30

**Session Information** **32**

**Bibliography** **34**

# List of Figures


1 Distribution of the age of the participants . . . . . 7
2 Amplitude distribution . . . . . 9
3 Variability of Amplitude according to experimental conditions . . . . . 10
4 Passive duration distribution . . . . . 11
5 Frequency of Self-turn errors . . . . . 12
6 Distributions of Self-turn errors . . . . . 13
7 Parameters prior distributions . . . . . 17
8 First model selection . . . . . 20
9 Posterior parameter distributions and trace plots. . . . . . 23
9 Posterior parameter distributions and trace plots (cont.). . . . . . 24
10 Posterior predictive check . . . . . 26
11 Distributions of the predicted Self-turn error mean according to environment, perception, and amplitude . . . . . 28
12 Distributions of the predicted means of Self-turn error according to perception and delay . . 30


# List of Tables


1 Number of observations for each participant. . . . . . 8
2 Number of observations according to experimental conditions. . . . . . 8
3 Descriptive statistics . . . . . 12
4 Effects included in the initial model. . . . . . 16
5 Model selection . . . . . 20
6 Effects included in the selected model. . . . . . 21
7 Estimated parameters of the selected model . . . . . 22
8 Bayesian $R^2$ . . . . . 25
9 Evidence ratio in favour of the selected model . . . . . 27
10 Predicted Self-turn error mean according to experimental conditions . . . . . 28
11 Ratio of the Self-turn errors increase between 90 degrees and 180 degrees rotation . . . . . 29
12 Predicted Self-turn error mean according to perception and delay conditions . . . . . 30
13 Comparisons between perception and delay conditions . . . . . 31
14 Ratio of the Self-turn errors between the different conditions . . . . . 31

# 1 Introduction

In this report the statistical analyses of the article *"Integration of Vision and Proprioception Facilitates Encoding but not Storage: an Immersive Virtual Reality Study"* are presented. The aim of the study was to investigate whether integration of vision and proprioception is beneficial for encoding the movement (i.e. the rotation) and if an advantage of this integration is also evident during the storage phase. To this end, sensory conditions, environment conditions, movement amplitude, and recall delay were manipulated.

Here, we focus only on the statistical analysis. For theoretical aspects, aims and hypotheses, experimental design, and interpretation of the results, the reader can refer directly to the manuscript.

## 1.1 Study summary

Participants completed a self-turning task. Using a customized swivel chair, participants were rotated by one of the experimenters and were then asked to rotate themselves back to their starting position. The angle reproduction error produced by the participant, computed as the absolute rotation difference between the starting position and the final position, was considered as the outcome variable to evaluate participants performance.

Each participant performed the task in different sensory and environment conditions. In particular, participans completed the task in a real environment (`R`) and in Immersive Virtual Reality (`IVR`). In each of these two environments, one blind condition removed all visual information such that only proprioceptive information could be used (`P` condition). Another condition limited the access to visual landmarks (removing visual information about the body and corners of the room while retaining the overall use of vision) in order to disrupt proprioception (`V` condition). Finally, there was a condition where reliable visual and proprioceptive information were available (`VP` condition).

Moreover, to control whether the amplitude and recall delay would affect performance, each condition was completed with two rotation amplitudes (approximately 90 and 180 degrees) and with three delays (0, 3, and 6 seconds) respectively.

## 1.2 Supplemental material

The Supplemental material is divided into different sections:

- **Section 2:** the statistical approach and the plan of analysis are presented.
- **Section 3:** the dataset is presented with a brief description of each variable.
- **Section 4:** the descriptive statistics are presented.
- **Section 5:** the model selection procedure is presented.
- **Section 6:** the selected model is presented.

# 2 Statistical Approach

In this section, the statistical approach and the plan of the analysis are presented. First, the Bayesian model selection approach is described. Subsequently, we discuss the reasons why Bayesian Generalized Mixed-Effects Models are used. Finally, the plan of the analysis is summarized.

## 2.1 Bayesian model selection

Bayesian model selection approach is used to explore which variables and interactions influence accuracy in the self-turning task.

- **Bayesian** refers to the fact that analysis is performed in a Bayesian framework. Bayesian statistical approach is presented in the next section (see Section 2.2).
- **Model selection** is a widely used exploratory approach that allows to identify the set of variables related to the outcome of interest. Starting from the most general model that encompasses all the variables and interactions of interest, at each step predictors are removed according to some criteria until a "preferred" model is obtained. In the present analysis, models are evaluated according to information criteria (Wagenmakers & Farrell, 2004; Yamashita et al., 2007). At each step, predictors are removed if the resulting model is better than the previous model according to information criteria.
- **Information criteria** provide an estimate of the average deviance (i.e. error) of the model's ability to predict new data, penalizing for model complexity (i.e., number of parameters; McElreath, 2016). Therefore, information criteria allow to evaluate models considering the trade-off between parsimony and goodness-of-fit (Vandekerckhove et al., 2015; Wagenmakers & Farrell, 2004): as the complexity of the model increases (i.e. more parameters) the fit to the data increases as well but generalizability (i.e. ability to predict new data) decreases. The researcher's aim is to find the right balance between fit and generalizability in order to describe, with a statistical model, the important feature of the studied phenomenon and not the random noise of the observed data. In the present analysis, the Watanabe-Akaike Information Criterion (WAIC; Gelman et al., 2014; Piironen & Vehtari, 2017; Vehtari et al., 2017) was used as the information criterion to evaluate the models. WAIC is the corresponding Bayesian version of the commonly used Akaike information criterion (AIC; Akaike, 1973). In line with the Bayesian approach (see Section 2.2), WAIC has the desirable property of averaging over the posterior distribution rather than conditioning on a point estimate. Lower WAIC values are interpreted as indicators of a better model (as with all the information criteria).

In this exploratory analysis, Bayesian model selection approach allows us to identify the "preferred" model that offers a parsimonious and "good enough" fit for the data.

## 2.2 Bayesian generalized mixed-effects models

Given the complex structure of the data, Bayesian generalized mixed-effects models are used. Specifically, data are characterized by: (1) a continuous non-normally distributed dependent variable (i.e. rotation error); (2) a within-subject factor (i.e. delay); (3) within-subject factors (i.e. perception and environment); (4) a quantitative independent variable (i.e. rotation amplitude).

- **Mixed-effects models** allow to take into account the repeated measures design of the experiment (i.e. observations nested within subjects). Thus, participants are treated as random effects, with random intercepts that account for interpersonal variability, whereas other variables were considered as fixed effects.

- **Generalized linear models** are used considering the Gamma distribution, with logarithmic link function, as the probability distribution of the dependent variable. Generalized linear models allow to model non-normally distributed data using appropriate probability distributions that reflect the characteristics of the data (Fox, 2016). Selecting an appropriate probability distribution provides better fit to the data and more reliable results (Lo & Andrews, 2015). Gamma distribution is advised in the case of positively skewed, non-negative data, when the variances are expected to be proportional to the square of the means (Ng & Cribbie, 2017). These conditions are respected by our dependent variable (see Section 4.5): only positive values were possible, with a positive skewed distribution, and we expected a greater variability of the possible results as the model predicted mean increases (i.e. a greater dispersion of participants' scores when greater mean values are predicted by the model).

- **Bayesian approach** is a valid alternative to the traditional frequentist approach (Gelman et al., 2013; Kruschke & Liddell, 2018) and in the case of mixed-effects models it allows for accurate estimation of complex models that would otherwise fail to converge (i.e. unreliable results) in a traditional frequentist approach (Bolker et al., 2009; Fong et al., 2010). Without going into philosophical reasons, which are beyond the scope of the present work (if interested consider Romeijn & van de Schoot, 2008), Bayesian inference has some unique elements that make the meaning and interpretation of the results different from the classical frequentist approach (Etz & Vandekerckhove, 2018). In particular, in the Bayesian approach, parameters are estimated using probability distributions (i.e. a range of possible values) and not a single point estimate (i.e. a single value). Bayesian inference has three main ingredients (van de Schoot et al., 2014): (1) *Priors*, the probability distributions of possible parameter values considering the information available before conducting the experiment; (2) *Likelihood*, the information given by the observed data about the probability distributions of possible parameter values; (3) *Posteriors*, the resulting probability distributions of possible parameter values, obtained by combining Priors and Likelihood through Bayes' Theorem. As a result, the Bayesian approach assesses the variability (i.e. uncertainty) of parameter estimates and provides associated

inferences via 95% Bayesian Credible Intervals (BCIs): the range of most credible parameter values given the prior distribution and the observed data. Thus, the Bayesian approach allows for the description of the phenomenon of interest through probabilistic statements, rather than a series of simplified reject/not-reject dichotomous decisions typically used in the null hypothesis significance testing approach (McElreath, 2016).

### 2.3 Plan of the analysis

Analysis are conducted with the R software version 4.0.2 (R Core Team, 2018). First, descriptive statistics are presented in Section 4 to observe the main characteristics of the collected data. Number of observations, summary statistics, and graphical representations of the variables of interest are reported according to experimental conditions. Moreover, the correlation between amplitude of the passive rotation and duration of the passive rotation is evaluated to assess consistency across trials (see Section 4.6).

After, the model selection procedure is conducted to evaluate the possible interactions between Self-turn error, environment, perception, delay, and amplitude (see Section 5).

Models are estimated using the R package `brms` (Bürkner, 2017) which is based on STAN programming language (Stan Development Team, 2017a, 2017b) and employs the No-U-Turn Sampler (NUTS; Hoffman & Gelman, 2014), an extension of Hamiltonian Monte Carlo (Neal, 2012). The basic idea of estimating parameters through a sampling procedure is to iteratively poll possible parameter values from pre-specified prior distributions. Parameter values that support the data are rewarded, whereas those values that do not support the data are penalized. After a large number of iterations, convergence upon the model parameters that optimally represent the data is obtained. Iterations of the estimation procedure are split among independent chains (i.e. independent sampling processes) to ensure that the model reliably converges on the same parameter values. Since each chain has a random starting point, it requires a certain number of iterations before reaching an optimal convergence. Thus, for each chain the initial samples, (i.e., *"warm-up"*), are discarded and only the subsequent samples are used for inference purposes.

The "preferred" model is presented in Section 6. Posterior distributions of the model parameters are reported. Model fit (i.e. the model's ability to explain the data) is evaluated using the alternative Bayesian definition of $R^2$ proposed by Gelman et al. (2018) (see Section 6.2) and qualitative visual inspection of the posterior predictive check (Kruschke, 2013). Moreover, the plausibility of effects is evaluated according to the evidence ratio between the selected model and the model without the effect considered (see Section 6.3). To interpret the effects, posterior predicted means are computed and represented graphically, together with Bayesian pairwise comparisons (i.e. predicted score differences between experimental conditions). Finally, to quantify the differences between experimental conditions, the effects are expressed as the ratio between the conditions of interest.

# 3 Data Presentation

The dataset was formed by 1723 observations on 14 variables.

1. **ID**. Categorical variable with the unique ID (from 1 to 48) of each participant.
2. **Age**. Numerical variable representing participants' age in years.
3. **Environment**. Categorical variable for the environmental conditions of the experiment. Levels of the variable are "`R`" for *Reality* and "`IVR`" for *Immersive Virtual Reality.*
4. **Perception**. Categorical variable for the sensory conditions of the experiment. Levels of the variable are "`P`" for *Proprioception*, "`V`" for *Vision*, and "`VP`" for *Vision + Proprioception.*
5. **Condition**. Categorical variable between 1 and 6 to specify the experimental condition.
   - *1* ) Reality and Proprioception
   - *2* ) Reality and Vision
   - *3* ) Reality and Vision + Proprioception
   - *4* ) IVR and Proprioception
   - *5* ) IVR and Vision
   - *6* ) IVR and Vision + Proprioception
6. **Delay**. Categorical variable (see Section 3.1) for the delay conditions of the experiment after which the rotation task was performed by the participants. Levels of the variable are `0 sec`, `3 sec`, and `6 sec`.
7. **Direction**. Categorical variable for the direction of the passive rotation imposed by the experimenter on the participant. Levels of the variable are "`R`" for *Right* and "`L`" for *Left.*
8. **Amplitude**. Categorical variable for the amplitude of the passive rotation imposed by the experimenter on the participant. Levels of the variable are "`Small`" for approximately 90 degrees and "`Big`" for approximately 180 degrees.
9. **Start_position**. Numerical variable representing the position from which the experimenter started the passive rotation.
10. **End_position**. Numerical variable representing the position where the experimenter stopped the passive rotation.
11. **Return_position**. Numerical variable representing the position in which the participant stopped the active self-turn.

12. **Angle**. Numerical variable representing the actual angle of the passive rotation imposed by the experimenter on the participant.

13. **Passive_duration**. Numerical variable representing the duration of the passive rotation in milliseconds.

14. **Error**. Numerical variable representing the absolute error committed by the participant in the Self-turn task, computed as the absolute rotation difference between *Start_Position* and *Return_Position*.

Below we report the structure of the dataset.

```
load(paste0(data_directory,"Dataset.rda"))

str(data)
```

```
## 'data.frame': 1723 obs. of  14 variables:
##  $ ID              : Factor w/ 48 levels "1","2","3","4",..: 1 1 1 1 1 1 1 1 1 1 ...
##  $ Age             : int  25 25 25 25 25 25 25 25 25 25 ...
##  $ Environment     : Factor w/ 2 levels "R","IVR": 2 2 2 2 2 2 1 1 1 1 ...
##  $ Perception      : Factor w/ 3 levels "P","V","VP": 2 2 2 2 2 2 3 3 3 3 ...
##  $ Condition       : Factor w/ 6 levels "1","2","3","4",..: 5 5 5 5 5 5 3 3 3 3 ...
##  $ Delay           : Factor w/ 3 levels "0","3","6": 2 2 3 3 1 1 3 3 2 2 ...
##  $ Amplitude       : Factor w/ 2 levels "Small","Big": 1 2 1 2 1 2 1 2 1 2 ...
##  $ Direction       : Factor w/ 2 levels "R","L": 1 2 1 1 1 2 2 1 1 2 ...
##  $ Start_position  : num  55 57 45 47 75.5 88 70 66.5 69 76 ...
##  $ End_position    : num  148 244 126 269 171 ...
##  $ Return_position : num  57 44 47 75.5 88 70.5 66.5 69 76 73 ...
##  $ Angle           : num  92.5 173.5 81 222 95.5 ...
##  $ Passive_duration: num  3300 4142 2516 4502 3268 ...
##  $ Error           : num  2 13 2 28.5 12.5 17.5 3.5 2.5 7 3 ...
```

## 3.1 Delay as categorical variable

Note that the variable Delay is considered as a categorical variable and not as an ordinal variable or a continuous variable. No definite hypothesis regarding the relationship between the delay and the error committed by the participant in the Self-turn task are available. Greater errors might be expected with the increasing of the delay but the type of interaction (i.e., linear or non linear) and possible thresholds above which it would be possible or not to observe the effect are unknown. Therefore, also given the limited number of the delay conditions, Delay is considered as a categorical variable.

# 4 Descriptive Statistics

## 4.1 Participants

48 participants participated to the study.

```
data%>%
  filter(!duplicated(ID))%>%
  nrow()
```

```
## [1] 48
```

### 4.1.1 Participants' age

Participants ranged from 18 to 26 years of age. The mean age of the participants is 21.6 years with a standard deviation of 2.4 The distribution of the age of participants is reported in Figure 1.

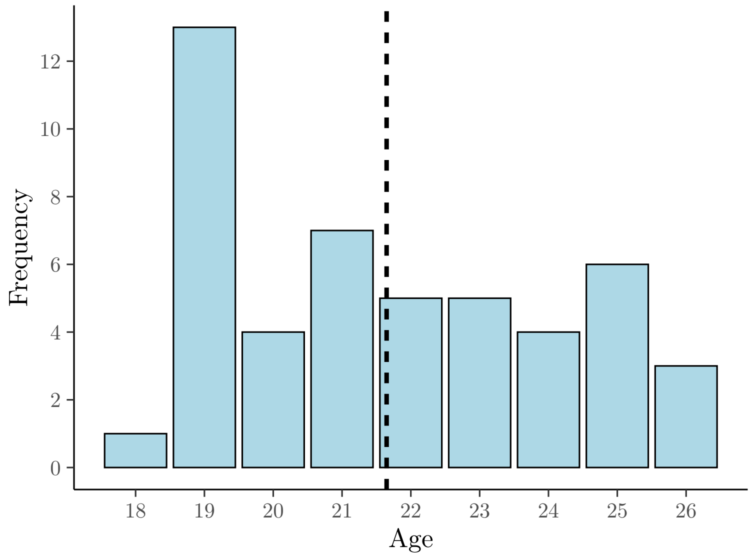


**Figure 1:** Distribution of the age of the participants ($n_{participants} = 48$). Dashed line represents the mean value ($Mean = 21.6$ years; $SD = 2.4$ years).

## 4.2 Number of observations

Out of the 48 participants, 43 participants completed the task in all 36 conditions and 5 participants completed 35 conditions (Table 1). Thus, the final dataset consists of 1723 observations nested in 48 participants.

**Table 1:** Number of observations for each participant.

| Number of participants | Number of observations |
|---|---|
| 5 | 35 |
| 43 | 36 |

*Note:* $n_{participants} = 48; n_{observations} = 1723$.

The number of observations according to experimental conditions is reported in Table 2.

**Table 2:** Number of observations according to experimental conditions.

| | Proprioception | Vision | Vision + Proprioception | Total |
|---|---|---|---|---|
| **Reality** | | | | |
| 0 sec | 96 | 96 | 97 | 289 |
| 3 sec | 96 | 95 | 94 | 285 |
| 6 sec | 95 | 96 | 96 | 287 |
| Total | 287 | 287 | 287 | 861 |
| **IVR** | | | | |
| 0 sec | 95 | 99 | 96 | 290 |
| 3 sec | 95 | 98 | 96 | 289 |
| 6 sec | 92 | 96 | 95 | 283 |
| Total | 282 | 293 | 287 | 862 |
| **Total** | | | | |
| 0 sec | 191 | 195 | 193 | 579 |
| 3 sec | 191 | 193 | 190 | 574 |
| 6 sec | 187 | 192 | 191 | 570 |
| Total | 569 | 580 | 574 | 1723 |

*Note:* IVR = Immersive Virtual Reality. $n_{participants} = 48; n_{observations} = 1723$

## 4.3 Amplitude

For each condition, the task aimed to include two rotations of different amplitude, one of approximately 180 degrees (`Big`) and the other approximately of 90 degrees (`Small`). However, given the variablity in the actual rotation (represented by `Angle`) reported in Figure 2, we consider Amplitude as a continuous variable and not as a dichotomous categorical variable.

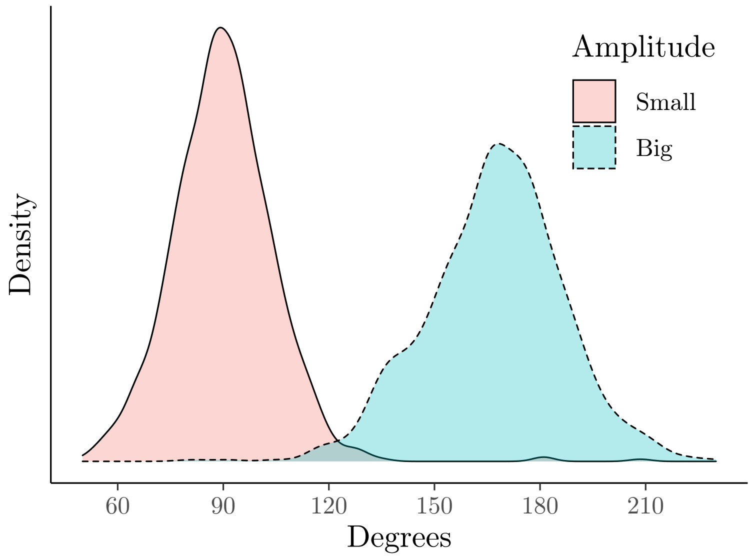


**Figure 2:** Amplitude distribution of the actual Self-turn rotation task (i.e. task difficulty; $n_{participants} = 48$; $n_{observations} = 1723$).

Moreover, to obtain interpretable results in the analyses, Amplitude values are standardized (i.e., Z-scores are obtained).

```
data<-data%>%
  mutate(Amplitude_st=(Angle-mean(Angle))/sd(Angle))
```

#### 4.3.1 Variability of the amplitude across conditions

In Figure 3 the distribution of the actual passive rotation Amplitude is presented according to the different experimental conditions. It is possible to observe that distributions are slightly different, but all of them cover appropriately the same range of values. Considering rotation amplitude as a continuous variable and not as a dichotomous categorical variable guarantees that results are not influenced by the variability in the actual rotation between the different experimental conditions.

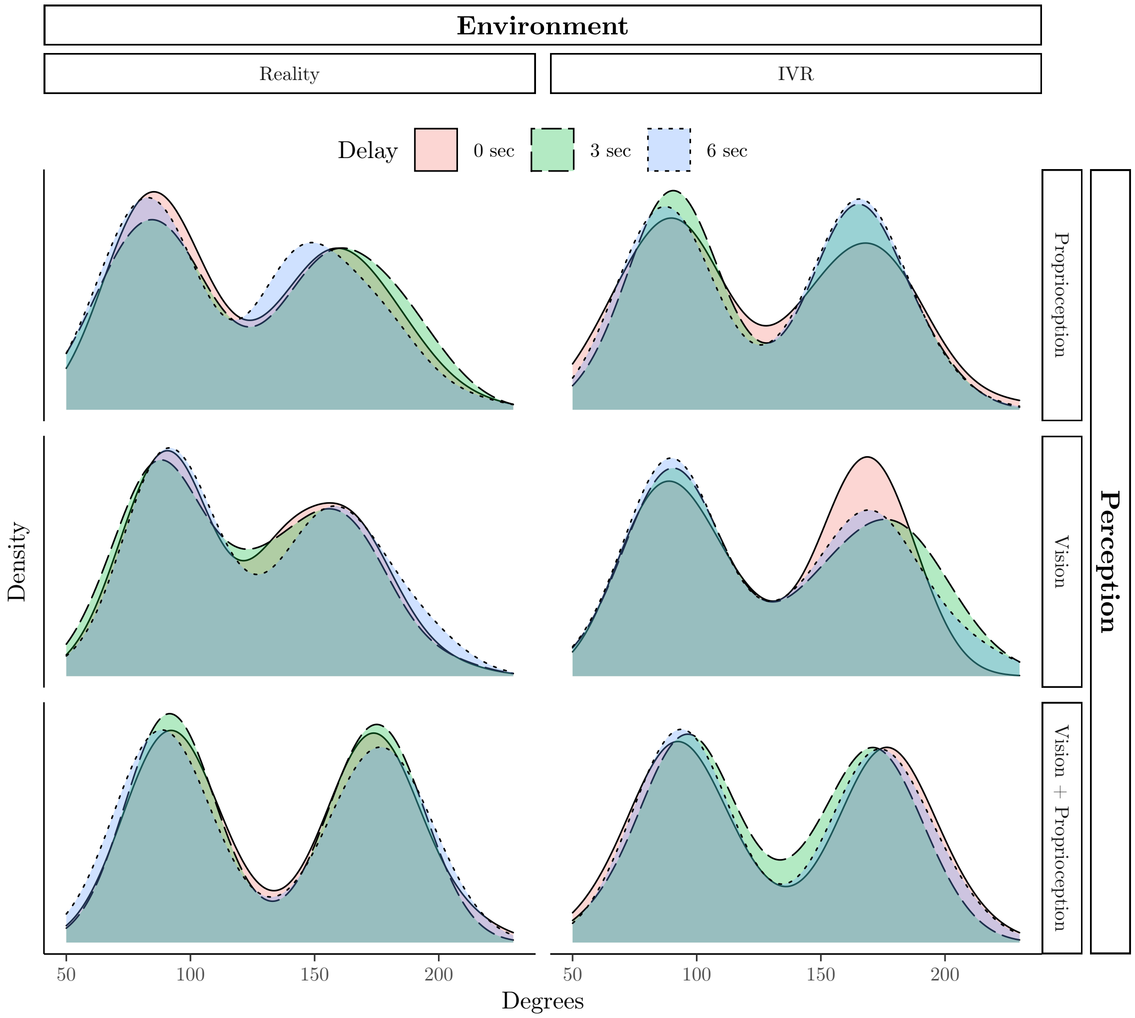


**Figure 3:** Amplitude distribution of the actual Self-turn rotation according to the different experimental conditions ($n_{participants} = 48$; $n_{observations} = 1723$).

## 4.4 Passive duration

In Figure 4 the distribution of the duration of the passive rotations are reported.

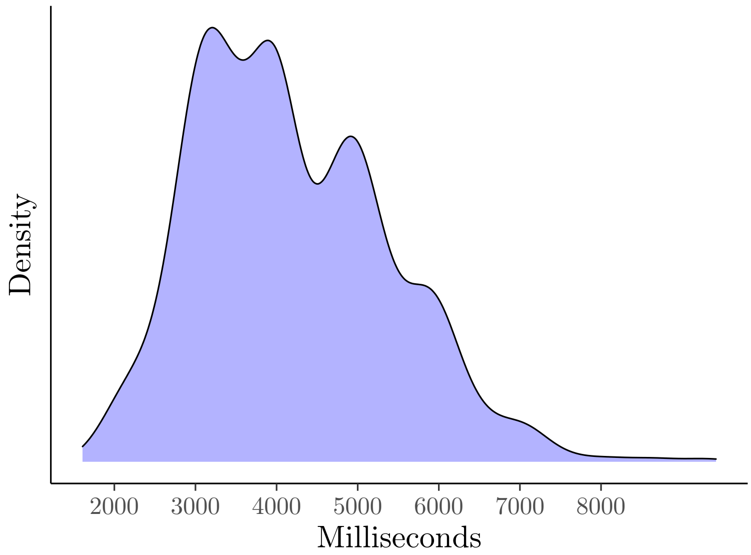


**Figure 4:** Distribution of the duration of the passive rotation in milliseconds ($n_{participants} = 48$; $n_{observations} = 1723$).

To obtain interpretable results in the analyses, Passive duration values are standardized (i.e., Z-scores are obtained).

```
data<-data%>%
  mutate(Passive_duration_st=(Passive_duration-mean(Passive_duration))/
           sd(Passive_duration))
```

### 4.5 Self-turn error

Given that observations are not independent but nested within participants, to compute descriptive statistics in each condition first the mean Self-turn error is obtained for each participant and then mean and standard deviation are computed.

In the present sample, the mean Self-turn error is 11.85 degrees ($SD = 3.26$).

```
with(data, mean(tapply(Error,INDEX=ID, FUN= mean)))

## [1] 11.85377

with(data, sd(tapply(Error,INDEX=ID, FUN= mean)))

## [1] 3.261765
```

Means and standard deviations according to the different experimental conditions are reported in Table 3.

**Table 3:** Descriptive statistics. Means and standard deviations of self-turn error according to experimental conditions

| | **Proprioception** | | **Vision** | | **Vision + Proprioception** | | **Total** | |
|---|---|---|---|---|---|---|---|---|
| | **Mean** | **SD** | **Mean** | **SD** | **Mean** | **SD** | **Mean** | **SD** |
| **Reality** | | | | | | | | |
| 0 sec | 16.6 | 9.8 | 6.3 | 5.2 | 5.0 | 4.1 | 9.3 | 4.0 |
| 3 sec | 17.5 | 12.1 | 5.0 | 3.9 | 5.4 | 3.4 | 9.5 | 5.6 |
| 6 sec | 17.4 | 11.1 | 5.4 | 3.4 | 5.8 | 5.5 | 9.5 | 4.6 |
| Total | 17.2 | 7.3 | 5.6 | 2.9 | 5.4 | 3.1 | 9.4 | 3.0 |
| **IVR** | | | | | | | | |
| 0 sec | 19.1 | 10.4 | 14.7 | 7.8 | 7.4 | 5.3 | 13.7 | 4.5 |
| 3 sec | 17.8 | 9.9 | 15.1 | 9.7 | 11.4 | 9.6 | 14.8 | 6.6 |
| 6 sec | 20.8 | 12.3 | 13.0 | 8.5 | 9.5 | 8.3 | 14.5 | 6.1 |
| Total | 19.1 | 8.1 | 14.3 | 6.6 | 9.4 | 5.9 | 14.3 | 4.9 |
| **Total** | | | | | | | | |
| 0 sec | 17.7 | 7.9 | 10.6 | 5.3 | 6.1 | 3.5 | 11.5 | 3.2 |
| 3 sec | 18.2 | 10.8 | 10.1 | 5.3 | 8.5 | 5.2 | 12.1 | 4.8 |
| 6 sec | 19.0 | 8.6 | 9.4 | 4.8 | 7.7 | 5.1 | 12.0 | 3.9 |
| Total | 18.3 | 6.5 | 10.0 | 3.8 | 7.4 | 3.4 | 11.9 | 3.3 |

*Note:* IVR = Immersive Virtual Reality. $n_{participants} = 48; n_{observations} = 1723$

The frequency of the observed values is reported in Figure 5. Since Self-turn error is computed as the absolute difference between `Start_position` and `Return_position`, only positive values are possible and from visual inspection the dependent variable has an evidently positive skewed distribution (skewness value = 2.26).

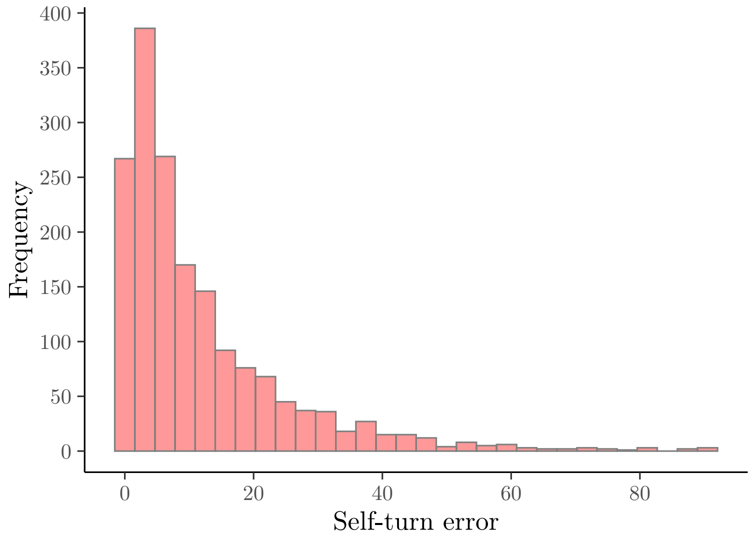


**Figure 5:** Frequency of the observed Self-turn errors ($n_{participants} = 48$; $n_{observations} = 1723$).

Finally, we represent in Figure 6 the distribution of the observed Self-turn error according to the different experimental conditions.

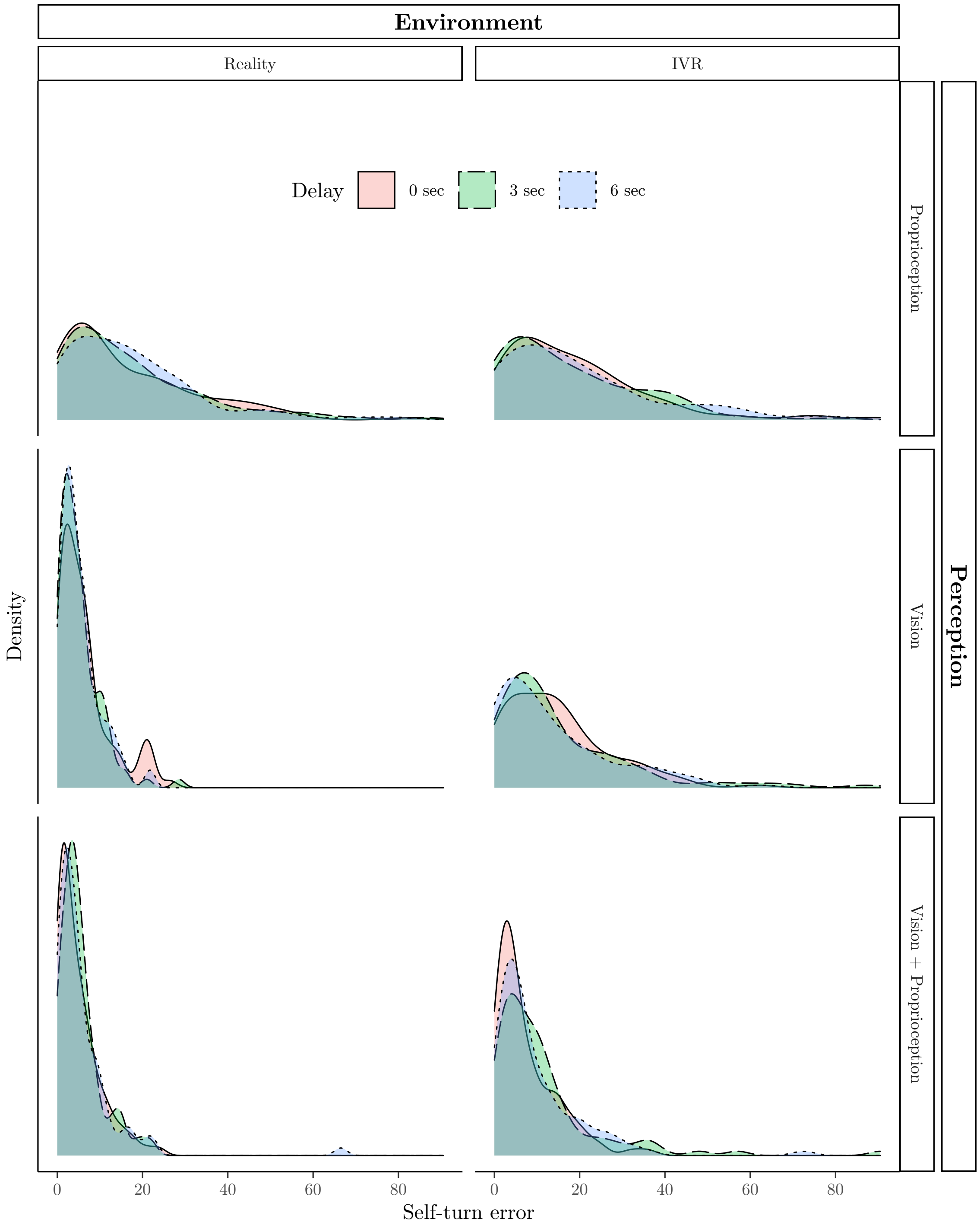


**Figure 6:** Distributions of the observed Self-turn errors according to the different experimental conditions ($n_{participants} = 48$; $n_{observations} = 1723$).

### 4.6 Correlation beetween amplitude and duration

Tp evaluate the consistency across trials, the correlation between amplitude and duration of thee passive rotation is evaluated.

```
cor.test(data$Amplitude_st, data$Passive_duration_st)
```

```
##
##  Pearson's product-moment correlation
##
## data:  data$Amplitude_st and data$Passive_duration_st
## t = 33.159, df = 1721, p-value < 2.2e-16
## alternative hypothesis: true correlation is not equal to 0
## 95 percent confidence interval:
##  0.5946739 0.6523531
## sample estimates:
##       cor
## 0.6243638
```

Results indicates a high positive correlation, $r(1721) = 0.62$ ($p < .001$), confirming a good consistency across trials.

# 5 Model Selection

In this section, the model selection evaluating the possible relations between Self-turn error, Environment, Perception, Delay, and Amplitude is presented. First, the initial model is described considering model definition and prior settings. Subsequently, the different steps of the model selection are reported.

## 5.1 Initial model

### 5.1.1 Generalized Linear Model

We specify the Gamma distribution with logarithmic link function as the family distribution for our Bayesian Generalized Mixed-Effects Models. Given that there are some 0 values in the Self-turn error, in order to use the logarithmic link function we need first to add 1 to all the errors. This is done to avoid indefinite values (i.e. $\log(0) = -\infty$).

```
data <- data%>%
  mutate(Error1=Error+1)
```

### 5.1.2 Model formula

The initial model considers the Self-turn error (`Error1`, see Section 5.1.1) as the dependent variable and as independent variables the environment of the experiment (`Environment`), the perception condition (`Perception`), the delay condition (`Delay`), the amplitude of the passive rotation (`Amplitude_st`, the standardized values are considered), and the direction of the rotation (`Direction`). Moreover, the participants (`ID`) are considered as a random effect on the intercept of the model, to account for baseline interpersonal variability.

In the model all the interactions between `Environment`, `Perception`, `Delay`, and `Amplitude_st` are considered up to the 4-way interaction. The initial model is presented below using R syntax where "$\sim$" stands for "is predicted by", "+" stands for additive effect,":" stands for interaction effect, "*" stands for factor crossing (i.e., all the interactions between variables are considered), and "(1|variable name)" stands for random effect on the intercept. Model formula:

$$\texttt{Error1} \sim \texttt{Environment} * \texttt{Perception} * \texttt{Delay} * \texttt{Amplitude\_st} + \texttt{Direction} + \texttt{(1|ID)}.$$

To illustrate all the interactions considered in the model, all the effects are reported in Table 4.

**Table 4:** Effects included in the initial model.

| | Effects | | |
|---|---|---|---|
| **Random Effect** | | | |
| | (1\|ID) | | |
| **Fixed Effects** | | | |
| *Main effects* | Environment | Perception | Delay |
| | Amplitude_st | Direction | |
| *2-way interactions* | Environment:Perception | Perception:Delay | |
| | Environment:Delay | Perception:Amplitude_st | |
| | Environment:Amplitude_st | Delay:Amplitude_st | |
| *3-way interactions* | Environment:Perception:Delay | | |
| | Environment:Perception:Amplitude_st | | |
| | Environment:Delay:Amplitude_st | | |
| | Perception:Delay:Amplitude_st | | |
| *4-way interaction* | Environment:Perception:Delay:Amplitude_st | | |

*Note:* ":" stands for interaction effect. "(1|variable name)" stands for random intercept

### 5.1.3 Models Priors

All models use default prior specification of the R package `brms` (Bürkner, 2017) that are considered to be only weakly informative in order to influence the results as little as possible, while providing at least some regularization to considerably improve convergence and sampling efficiency. This allows posterior distributions to be mostly influenced by the observed data rather than by prior information.

In particular, for the population-level effects (i.e. fixed effects) `brms` uses an improper flat prior over the reals. Group-level effects (i.e. random effects) are restricted to be non-negative and, by default, they are set to `student_t(3, 0, 2.5)`, that is a half student-t prior with 3 degrees of freedom and a scale parameter of 2.5. Intercept prior is set to `student_t(3, 2.1, 2.5)`, that is a student-t with 3 degrees of freedom, location parameter of 2.1 and scale parameter of 2.5. Finally the prior for the shape parameter for the gamma distribution is set to `gamma(0.01, 0.01)`. In Figure 7 the different prior distributions are presented.

Remember that by default, the population-level intercept is estimated separately and not as part of population-level parameter vector `b`. Moreover, to increase sampling efficiency, the population-level design matrix `X` is centered around its column means `X_means`. Thus, intercept prior is defined on the centered design matrix and not on the real intercept.

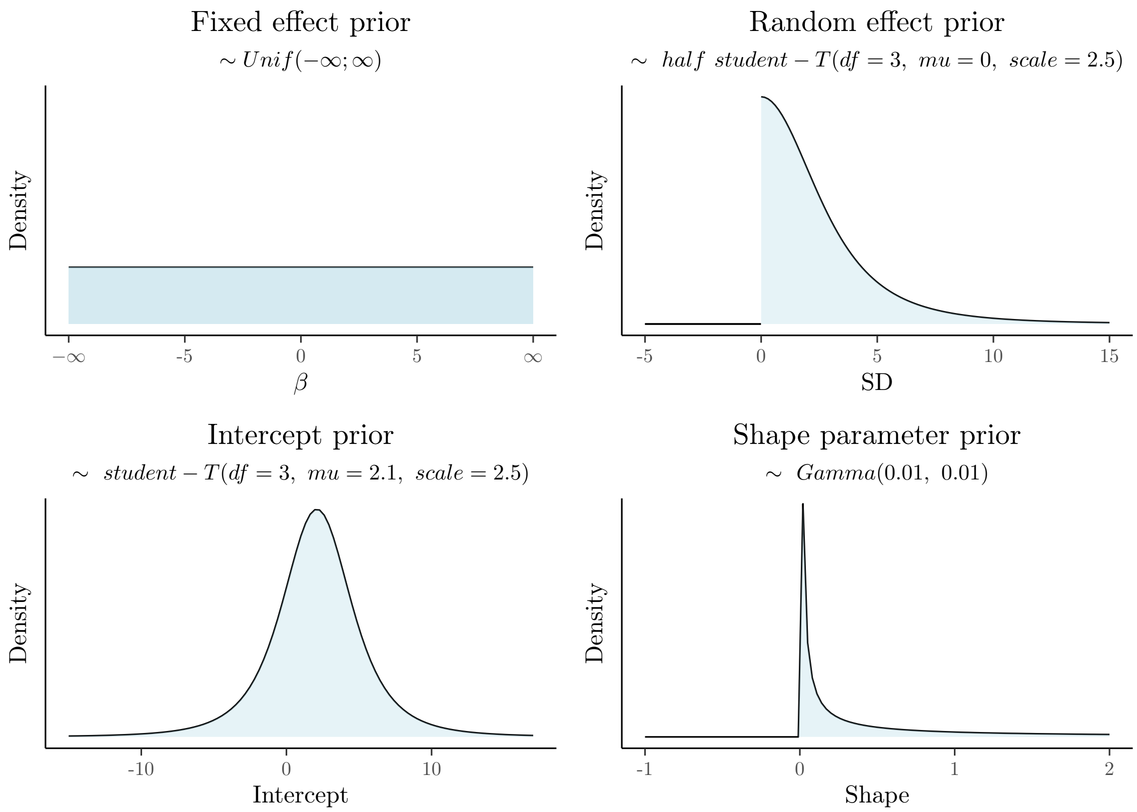


**Figure 7:** Parameter prior distributions

Below we report the default prior settings of brms for a simple model with one categorical predictor (i.e., `Direction`), one continuous predictor (i.e., `Amplitude_st`), and one random effect on the intercept (i.e., `(1|ID)`). For the other models the settings are the same just with more $\beta$ parameters.

```
get_prior(Error1~ Amplitude_st+Direction+(1|ID),family=Gamma(link="log"),data=data)
```

```
##                    prior     class         coef group resp dpar nlpar bound
##                   (flat)         b
##                   (flat)         b Amplitude_st
##                   (flat)         b   DirectionL
##  student_t(3, 2.1, 2.5) Intercept
##    student_t(3, 0, 2.5)        sd
##    student_t(3, 0, 2.5)        sd                  ID
##    student_t(3, 0, 2.5)        sd    Intercept     ID
##       gamma(0.01, 0.01)     shape
##        source
##       default
##  (vectorized)
##  (vectorized)
##       default
##       default
##  (vectorized)
##  (vectorized)
##       default
```

### 5.1.4 Models Estimation

Each model is estimated using 6 independent chains of 4,000 iterations with a *"warm-up"* period of 2,000 iterations, resulting in 12,000 usable samples. Convergence is evaluated considering R-hat diagnostic criteria, where values close to 1 indicate convergence and 1.100 is the proposed threshold for convergence (Gelman & Rubin, 1992). For each model, number of fixed-effects parameters, WAIC estimated value and standard error, maximum R-hat value, and minimum Effective Sample Size (ESS) are reported. WAIC values are considered to evaluate the models, where lower values represent better results. ESS is the number of actual independent samples used for inference (Stan Development Team, 2017a). An R function named `model_info` is defined to obtain the information of interest from the model.

```
model_info<-function(model){
  summary<-summary(model)$fixed
  waic<-waic(model)$estimates
  summary_model<-data.frame(Model=deparse(substitute(model)),
                            n_fixed_par=nrow(summary),
                            Waic_est=waic[3,1],
                            Waic_se=waic[3,2],
                            max_Rhat=max(summary[,5]),
                            min_Bulk_ESS=min(summary[,6]),
                            min_Tail_ESS=min(summary[,7]),
                            stringsAsFactors = F)
  return(summary_model)
}
```

## 5.2 Model selection steps

Starting from the initial model `m_0`, predictors are removed to obtain a better model according to the WAIC (i.e., lower WAIC values means better model). Each step in the model selection represents a new model which is better than the model in the previous step. The R code for the model selection procedure is reported below and model selection steps with relative information for all models are presented in Table 5. Relative WAIC weights are computed to assess the probability of each model to make the best prediction on new data, conditional on the set of models included in the model selection procedure (McElreath, 2016).

In Figure 8 model selection procedure is represented considering the WAIC values for each model.

```
set.seed(2019)

# Initial model
m_0<-brm(Error1~ Environment*Perception*Delay*Amplitude_st + Direction+(1|ID),
         data= data,chains = 6, warmup = 2000, iter = 4000, family=Gamma (link="log"), seed=2019)
m_0<-add_waic(m_0)

# Step 1
m_1<-update(m_0, formula. = ~ . - Environment:Perception:Delay:Amplitude_st,
            newdata =  data, warmup=2000, seed=2019)
```

```
m_1<-add_waic(m_1)

# Step 2
m_2<-update(m_1, formula. = ~ . - Perception:Delay:Amplitude_st,
            newdata =  data, warmup=2000, seed=2019)
m_2<-add_waic(m_2)

# Step 3
m_3<-update(m_2, formula. = ~ . - Environment:Delay:Amplitude_st,
            newdata =  data, warmup=2000, seed=2019)
m_3<-add_waic(m_3)

m_4<-update(m_3, formula. = ~ . - Environment:Perception:Amplitude_st,
            newdata =  data, warmup=2000, seed=2019)
m_4<-add_waic(m_4)

# Step 4
m_5<-update(m_3, formula. = ~ . - Environment:Perception:Delay,
            newdata =  data, warmup=2000, seed=2019)
m_5<-add_waic(m_5)

# Step 5
m_6<-update(m_5, formula. = ~ . - Delay:Amplitude_st,
            newdata =  data, warmup=2000, seed=2019)
m_6<-add_waic(m_6)

m_7<-update(m_6, formula. = ~ . - Perception:Delay,
            newdata =  data, warmup=2000, seed=2019)
m_7<-add_waic(m_7)

# Step 6
m_8<-update(m_6, formula. = ~ . - Environment:Delay,
            newdata =  data, warmup=2000, seed=2019)
m_8<-add_waic(m_8)

# Step 7
m_9<-update(m_8, formula. = ~ . - Direction,
            newdata =  data, warmup=2000, seed=2019)
m_9<-add_waic(m_9)

m_10<-update(m_9, formula. = ~ . - Perception:Delay
                                   - Delay,
            newdata =  data, warmup=2000, seed=2019)
m_10<-add_waic(m_10)
```

Model `m_9` has the lowest WAIC value.

**Table 5:** Model selection

| Steps | Parameters removed from previous step | Model | n fixed parameters | WAIC Estimate | WAIC SE | WAIC Weights | max R-hat | min ESS Bulk | min ESS Tail |
|---|---|---|---|---|---|---|---|---|---|
| Initial model | | m_0 | 37 | 11504.8 | 89.9 | 0.00 | 1.002 | 2102 | 4560 |
| Step 1 | Environment:Perception:Delay:Amplitude_st | m_1 | 33 | 11499.3 | 89.4 | 0.00 | 1.003 | 3391 | 5392 |
| Step 2 | Perception:Delay:Amplitude_st | m_2 | 29 | 11494.0 | 89.6 | 0.00 | 1.002 | 4198 | 6404 |
| Step 3 | Environment:Delay:Amplitude_st | m_3 | 27 | 11492.5 | 89.9 | 0.00 | 1.002 | 4485 | 6877 |
| | Environment:Perception:Amplitude_st | m_4 | 25 | 11500.8 | 90.0 | 0.00 | 1.002 | 4281 | 7083 |
| Step 4 | Environment:Perception:Delay | m_5 | 23 | 11485.9 | 89.8 | 0.01 | 1.001 | 4744 | 6803 |
| Step 5 | Delay:Amplitude_st | m_6 | 21 | 11482.0 | 89.7 | 0.06 | 1.001 | 4081 | 6442 |
| | Perception:Delay | m_7 | 17 | 11489.7 | 90.1 | 0.00 | 1.002 | 4437 | 6342 |
| Step 6 | Environment:Delay | m_8 | 19 | 11479.6 | 89.9 | 0.20 | 1.002 | 3519 | 6048 |
| Step 7 | Direction | m_9 | 18 | 11477.3 | 89.9 | 0.64 | 1.002 | 4000 | 6749 |
| | Perception:Delay and Delay | m_10 | 12 | 11481.2 | 90.3 | 0.09 | 1.001 | 4117 | 6452 |

*Note:* ESS = Effective Sample Size

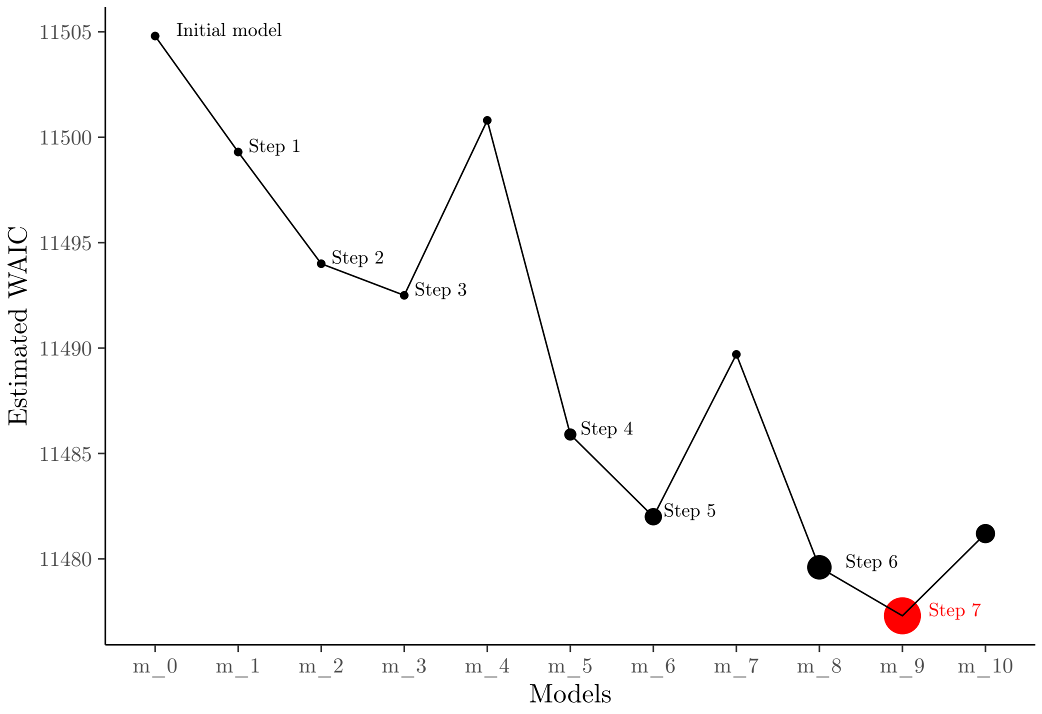


**Figure 8:** Model selection. Point sizes are proportional to the WAIC weights.

# 6 First Model Presentation

Model m_9 is selected as the "preferred" model by the model selection procedure at step 7. The selected model has the probability of being the best model of 0.64 that is superior to all the other models (m_8 has a probability of 0.2 and all the others models have probabilities lower than 0.09).

```
selected_model<-m_9
```

In this section, first, the estimated parameters of the selected model are reported. Then, the model fit is evaluated considering $R^2$ value and model Posterior Predictive check. Main effects and interaction effects are interpreted using graphical representations of the predicted values by the model. Finally, planned comparison and effects sizes are presented.

## 6.1 Selected model

The selected model includes the 3-way interaction between Environment, Perception, and Amplitude_st, the 2-way interaction between Perception and Delay, and the random intercept (1|ID). The formula of the selected model is:

$$\texttt{Error1} \sim \texttt{Environment} * \texttt{Perception} * \texttt{Amplitude\_st} + \texttt{Perception} * \texttt{Delay} + \texttt{(1|ID)}$$

To illustrate all the interactions considered in the model, all the effects are reported in Table 6.

**Table 6:** Effects included in the selected model.

| | Effects | |
|---|---|---|
| **Random Effect** | | |
| | (1\|ID) | |
| **Fixed Effects** | | |
| *Main effects* | Environment | Perception |
| | Delay | Amplitude_st |
| *2-way interactions* | Environment:Perception | |
| | Environment:Amplitude_st | |
| | Perception:Amplitude_st | |
| | Perception:Delay | |
| *3-way interaction* | Environment:Perception:Amplitude_st | |

*Note:* ":" stands for interaction effect. "(1|variable name)" stands for random intercept

### 6.1.1 Parameter posterior distributions

The 95% Bayesian Credible Intervals (BCIs) of the parameter posterior distributions are reported in Table 7. The 95% BCIs represent the range of the 95% most credible parameter values given the prior distribution and the observed data. Posterior parameter distribution and trace plots are presented in Figure 9

**Table 7:** Estimated parameters of the selected model

| Parameters | Estimate | Est.Error | 95 % BCI Lower | 95 % BCI Upper | Rhat | Eff.Sample Bulk | Eff.Sample Tail |
|---|---|---|---|---|---|---|---|
| **Random Effects** | | | | | | | |
| sd(Intercept) | 0.22 | 0.03 | 0.17 | 0.29 | 1.001 | 4693 | 7079 |
| **Main Effect** | | | | | | | |
| Intercept | 2.87 | 0.07 | 2.73 | 3.01 | 1.002 | 4000 | 6761 |
| EnvironmentIVR | 0.04 | 0.06 | -0.09 | 0.16 | 1.001 | 6338 | 8491 |
| PerceptionV | -0.92 | 0.09 | -1.09 | -0.74 | 1.001 | 4712 | 7308 |
| PerceptionVP | -1.17 | 0.09 | -1.35 | -0.99 | 1.002 | 4671 | 6847 |
| Delay3 | -0.02 | 0.08 | -0.18 | 0.13 | 1.000 | 4778 | 7446 |
| Delay6 | 0.06 | 0.08 | -0.10 | 0.21 | 1.002 | 4708 | 6749 |
| Amplitude_st | 0.35 | 0.05 | 0.26 | 0.44 | 1.001 | 4620 | 6914 |
| **2-way interactions** | | | | | | | |
| EnvironmentIVR:PerceptionV | 0.72 | 0.09 | 0.54 | 0.89 | 1.001 | 7140 | 9067 |
| EnvironmentIVR:PerceptionVP | 0.38 | 0.09 | 0.21 | 0.56 | 1.001 | 7029 | 8591 |
| PerceptionV:Delay3 | -0.08 | 0.11 | -0.29 | 0.13 | 1.001 | 5196 | 7440 |
| PerceptionVP:Delay3 | 0.25 | 0.11 | 0.04 | 0.47 | 1.001 | 5375 | 7192 |
| PerceptionV:Delay6 | -0.20 | 0.11 | -0.42 | 0.01 | 1.001 | 5071 | 7329 |
| PerceptionVP:Delay6 | 0.14 | 0.11 | -0.07 | 0.36 | 1.001 | 4839 | 7602 |
| EnvironmentIVR:Amplitude_st | 0.05 | 0.06 | -0.08 | 0.17 | 1.001 | 4513 | 6796 |
| PerceptionV:Amplitude_st | -0.33 | 0.07 | -0.47 | -0.20 | 1.000 | 5467 | 7316 |
| PerceptionVP:Amplitude_st | -0.35 | 0.06 | -0.47 | -0.22 | 1.001 | 5082 | 7507 |
| **3-way interactions** | | | | | | | |
| EnvironmentIVR: PerceptionV:Amplitude_st | 0.33 | 0.09 | 0.15 | 0.51 | 1.000 | 5123 | 7008 |
| EnvironmentIVR: PerceptionVP:Amplitude_st | 0.14 | 0.09 | -0.03 | 0.32 | 1.001 | 5203 | 7615 |
| **Family Specific Parameter** | | | | | | | |
| shape | 1.75 | 0.06 | 1.64 | 1.86 | 1.001 | 16362 | 8864 |

*Note:* Baseline category for Environment is reality (texttttR). Baseline category for Perception is proprioception (texttttP). Baseline category for Delay is texttt0 seconds. IVR = Immersive Virtual Reality. V = Vision. VP = Vision + Proprioception. $n_{participants} = 48; n_{observations} = 1723$

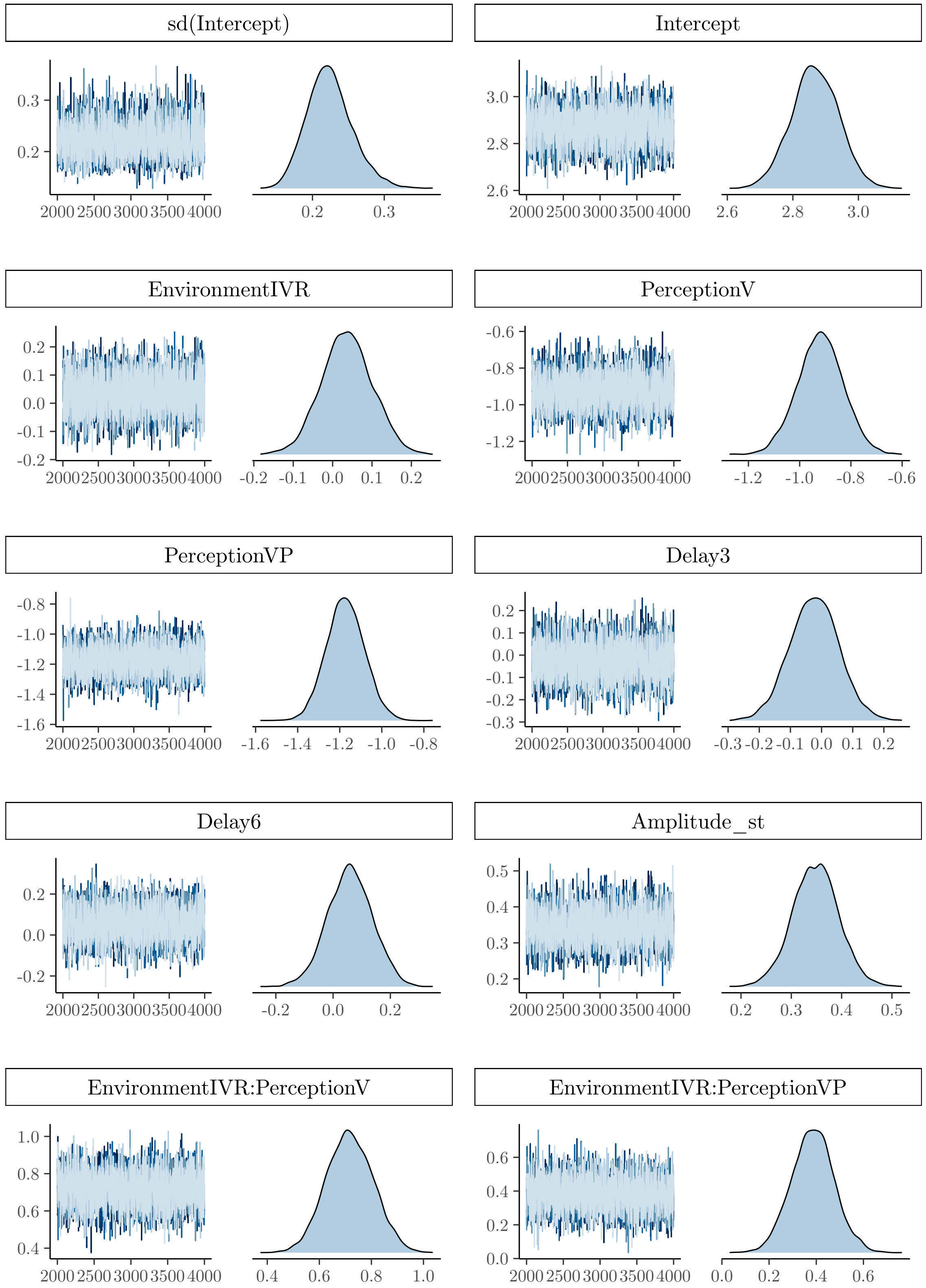


**Figure 9:** Posterior Parameter distributions and trace plots.

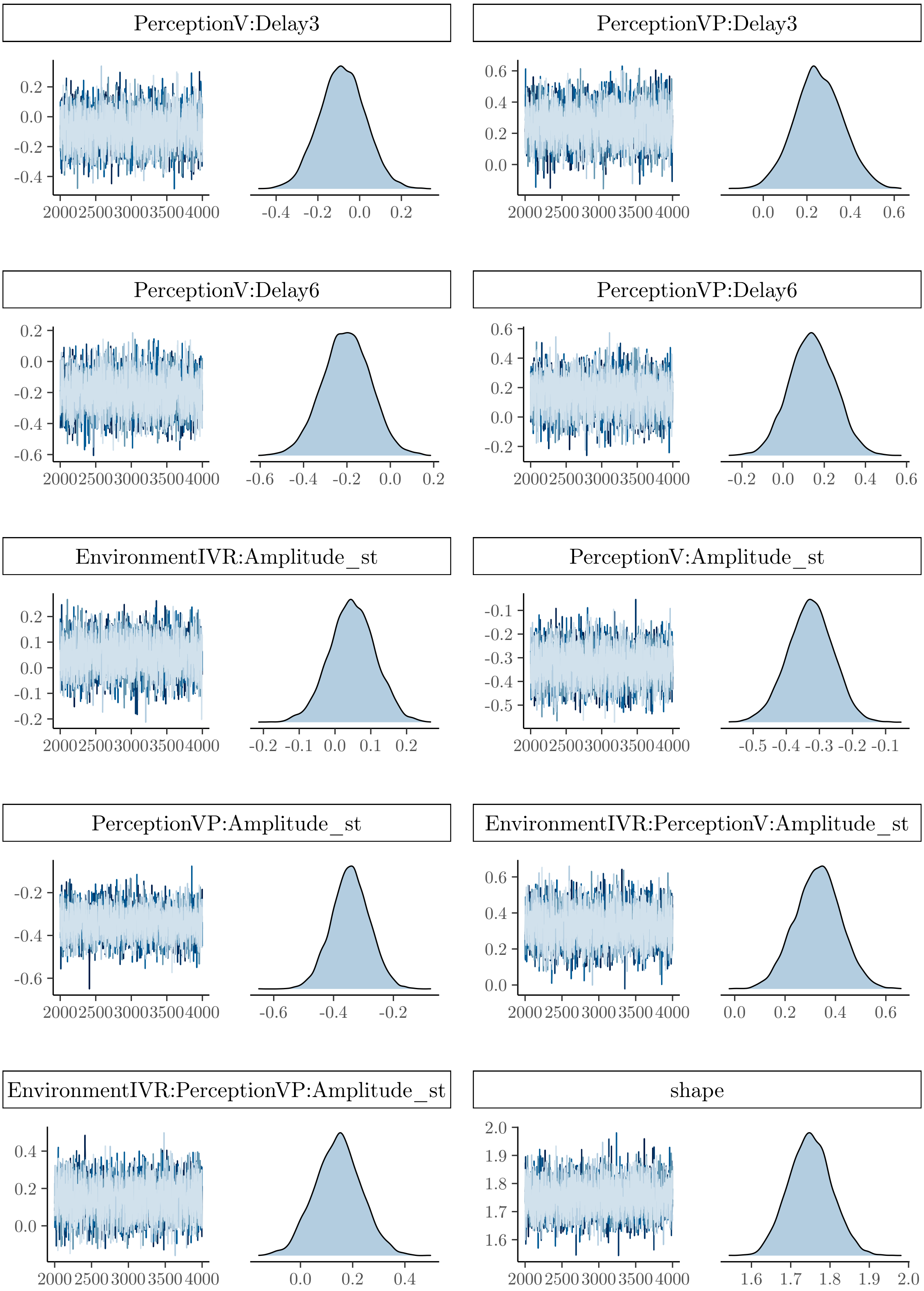


**Figure 9:** Posterior Parameter distributions and trace plots (cont.).

## 6.2 Model Fit

Commonly, the model fit (i.e. the model's ability to explain the data) is assessed using statistical indexes as $R^2$ (Xu, 2003). However, the usual definition of $R^2$ (i.e. the variance by the model divided by the total variance of the data) is problematic within the Bayesian framework since the numerator can be larger than the denominator (Gelman et al., 2018). To overcome this issue, Gelman et al. (2018) proposed a Bayesian alternative definition of $R^2$:

$$Bayesian\ R^2 = \frac{Explained\ Variance}{Explained\ Variance + Residual\ Variance} = \frac{V_{n=1}^{N}\hat{y}_n}{V_{n=1}^{N}\hat{y}_n + V_{n=1}^{N}r_n}, \qquad (1)$$

where $r_n = y_n - \hat{y_n}$ are the residuals of the fitted model. Given this definition, Bayesian $R^2$ is always between 0 and 1 by construction, and thus it follows the same interpretation as the common $R^2$: an estimate of the proportion of variance explained.

The estimated value of Bayesian $R^2$ for the selected model is 0.35 ($95\% BCI = 0.3; 0.4$), which means that the model explains 35% of the variability of the data (Table 8).

**Table 8:** Bayesian $R^2$

| **Bayesian $R^2$** | | **95 % BCI** | |
|---|---|---|---|
| **Estimate** | **SE** | **Lower** | **Upper** |
| 0.351 | 0.024 | 0.303 | 0.399 |

*Note:* $n_{participants} = 48; n_{observations} = 1723$

Posterior predictive check is an alternative way to evaluate the goodness-of-fit of a model (Kruschke, 2013). Qualitative visual inspection of the posterior predictive check allows to assess whether data predicted by the model resemble the observed data or if the residuals between actual and simulated data follow certain patterns.

In Figure 10 the data predicted by 100 sampled parameter vectors are presented. Visual inspection reveals a good fit of the model with the predicted data that properly and consistently follow the observed data.

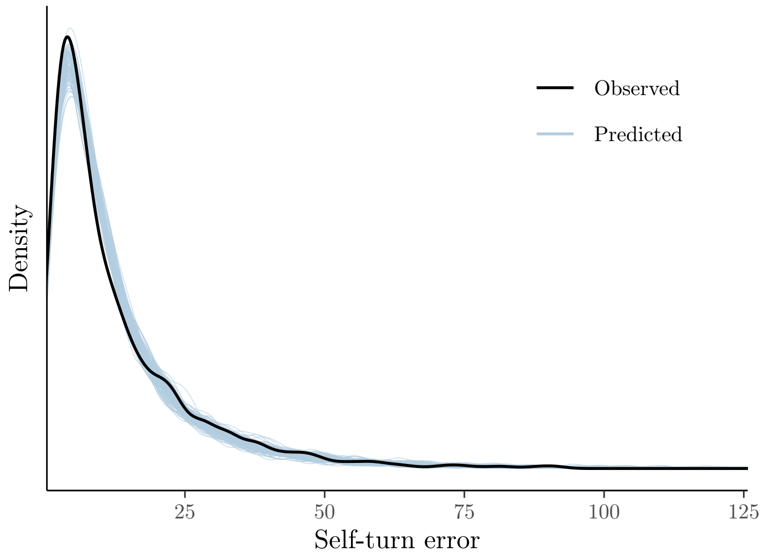


**Figure 10:** Posterior predictive check. The black line represents the distribution of observed data ($n_{participants} = 48$; $n_{observations} = 1723$). The gray lines represent the distribution of predicted data for each of the sampled parameter vector ($n_{samples} = 100$).

### 6.3 Effects evidence ratio

To evaluate the relative plausibility of the 3-way interaction between `Environment`, `Perception`, and `Amplitude_st` and the 2-way interaction between `Perception` and `Delay`, the evidence ratios between the selected model and the models without the effects of interest are considered.

```
# Model without 3 way interaction between Environment:Perception:Amplitude_st
m_no_3way <- update(selected_model, formula. = ~ . - Environment:Perception:Amplitude_st,
                              newdata =  data, warmup=2000, seed=2019)
m_no_3way <- add_waic(m_no_3way)

# Model without 2 way interaction between Perception:Delay
m_no_2way <- update(selected_model, formula. = ~ . - Perception:Delay,
                             newdata =  data, warmup=2000, seed=2019)
m_no_2way <- add_waic(m_no_2way)
```

The evidence ratio is computed as the exponential of minus the difference between the two models WAIC values divided by 2. That is,

$$\text{Evidence Ratio} = exp(\frac{-\Delta_{WAIC}}{2}),$$

where $\Delta_{WAIC} = WAIC_{\text{selected model}} - WAIC_{\text{compared model}}$. The evidence ratios are reported in Table 9.

**Table 9:** Evidence ratio in favour of the selected model

| Compared model | Effect removed | WAIC | Evidence ratio |
|---|---|---|---|
| m_no_3way | Environment:Perception:Amplitude_st | 11486.12 | 83 |
| m_no_2way | Perception:Delay | 11485.02 | 48 |

*Note:* $n_{participants} = 48; n_{observations} = 1723$

The selected model is 83 times more likely than the model without the 3-way interaction between environment, perception, and rotation amplitude, and 48 times more likely than the model without the 2-way interaction between perception and delay.

### 6.4 Posterior effect distributions

In order to interpret the results, the posterior parameter distributions are used to compute the predicted distributions of Self-turn error means considering the experimental conditions of interest and marginalizing for the other variables (i.e., averaging over the other variables). To obtain results on the original scale, the value 1 is subtracted from the predicted values (see section5.1.1).

The predicted values are computed using the R function `fitted()` that does not include the residual (observation-level) variance and `re_formula = NA` is declared to not include the random effect of participants `ID`.

```
# Create all experimental conditions
post_data=expand.grid(Environment=c("R","IVR"),
                      Perception=c("P","V","VP"),
                      Delay=c("0","3","6"),
                      Amplitude=seq(from=min(data$Angle), to=max(data$Angle),by = 10))%>%
  mutate(Amplitude_st=(Amplitude-mean(data$Angle))/sd(data$Angle))%>%

  # Predicted values (and remove 1)
  cbind(.,t(fitted(selected_model, newdata = .,re_formula = NA,summary = FALSE)-1))%>%
  pivot_longer(cols="1":"12000", names_to = "iter", values_to = "value",)%>%
  mutate(iter=as.numeric(iter))
```

#### 6.4.1 Interaction `Environment:Perception:Amplitude_st`

The predicted Self-turn error mean according to Environment, Perception, and Amplitude of the passive rotation is presented in Figure 11. The predicted values at 90 degrees and 180 degrees of Amplitude are reported in Table 10.

```
# Marginalize over other conditions
Interaction_3way <- post_data%>%
  group_by(Environment,Perception,Amplitude,iter)%>%
  summarize(value=mean(value))
```

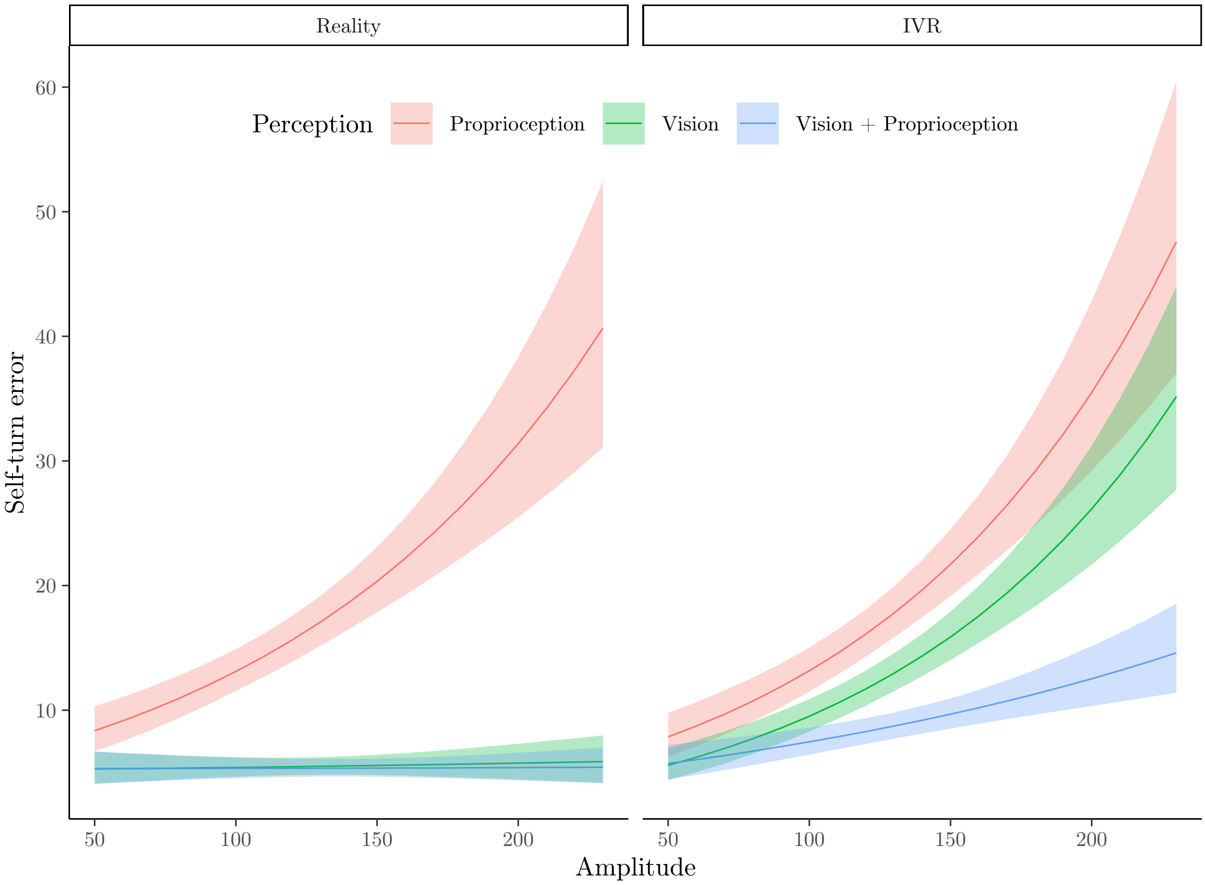


**Figure 11:** Distributions of the predicted Self-turn error mean according to environment, perception, and amplitude ($n_{participants} = 48$; $n_{observations} = 1723$).

**Table 10:** Predicted Self-turn error mean according to experimental conditions

| **Conditions** | | | **Error** | **95 % BCI** | |
|---|---|---|---|---|---|
| **Environment** | **Amplitude** | **Perception** | **Mean** | **Lower** | **Upper** |
| **Reality** | 90 | Proprioception | 11.99 | 10.42 | 13.79 |
| | | Vision | 5.38 | 4.56 | 6.29 |
| | | Vision + Proprioception | 5.32 | 4.48 | 6.26 |
| | 180 | Proprioception | 26.40 | 22.25 | 31.19 |
| | | Vision | 5.67 | 4.60 | 6.90 |
| | | Vision + Proprioception | 5.37 | 4.50 | 6.35 |
| **IVR** | 90 | Proprioception | 11.89 | 10.24 | 13.74 |
| | | Vision | 8.57 | 7.35 | 9.93 |
| | | Vision + Proprioception | 7.08 | 6.01 | 8.29 |
| | 180 | Proprioception | 29.17 | 24.81 | 34.09 |
| | | Vision | 21.44 | 18.33 | 24.93 |
| | | Vision + Proprioception | 11.30 | 9.64 | 13.23 |

*Note:* IVR = Immersive Virtual Reality. $n_{participants} = 48; n_{observations} = 1723$

To quantify the relative increase in the different conditions, the ratios of the Self-turn errors between 180-degrees and 90-degrees Amplitude are reported in Table 11.

In the reality environment, participants are expected to make greater Self-turn errors with increasing Amplitude only in the Proprioception condition. On the contrary, in the Vision and Vision+Proprioception conditions the Self-turn error remains the same. In the IVR environment, greater Self-turn errors with increasing Amplitude are expected in all conditions. However, the increase is smaller in the Vision+Proprioception condition and similar in the Proprioception and the Vision conditions.

**Table 11:** Ratio of the Self-turn errors increase between 90 degrees and 180 degrees rotation

| Condition | | | Ratio | 95 % BCI | |
|---|---|---|---|---|---|
| **Amplitude** | **Environment** | **Perception** | **Mean** | **Lower** | **Upper** |
| **180 / 90 degrees** | Reality | Proprioception | 2.21 | 1.79 | 2.70 |
| | | Vision | 1.06 | 0.82 | 1.36 |
| | | Vision + Proprioception | 1.02 | 0.81 | 1.26 |
| | IVR | Proprioception | 2.47 | 2.01 | 2.99 |
| | | Vision | 2.51 | 2.06 | 3.05 |
| | | Vision + Proprioception | 1.61 | 1.30 | 1.97 |

*Note:* IVR = Immersive Virtual Reality. $n_{participants} = 48; n_{observations} = 1723$

Below, Bayesian pairwise comparisons of interest (i.e. predicted score differences between conditions) are presented:

- `IVR_P_180-IVR_V_180`. In the IVR environment considering 180 degrees rotation amplitude, participants are expected to make greater Self-turn errors in the Proprioception condition than in the Vision condition (Mean = 7.73 degrees; 95% BCI=2.63; 13.02).

- `IVR_P_180-IVR_VP_180`. In the IVR environment considering 180 degrees rotation amplitude, participants are expected to make greater Self-turn errors in the Proprioception condition than in the Vision+Proprioception condition (Mean = 17.87 degrees; 95% BCI=13.5; 22.82).

- `IVR_V_180-IVR_VP_180`. In the IVR environment considering 180 degrees rotation amplitude, participants are expected to make greater Self-turn errors in the Vision condition than in the Vision+Proprioception condition (Mean = 10.14 degrees; 95% BCI=6.81; 13.73)

- `(IVR_VP_180-IVR_VP_90) - (R_VP_180-R_VP_90)`. The expected difference between the difference considering 180 and 90 degrees rotation in the IVR environment Vision+Proprioception condition and the difference considering 180 and 90 degrees rotation in the reality environment Vision+Proprioception condition is 4.18 degrees (95% BCI=1.9; 6.56).

### 6.4.2 Interaction `Perception:Delay`

The predicted Self-turn error mean according to Perception and Delay is presented in Figure 12 and values are reported in Table 12.

```
# Marginalize over other conditions
Interaction_2way <- post_data%>%
  group_by(Perception,Delay,iter)%>%
  summarize(value=mean(value))
```

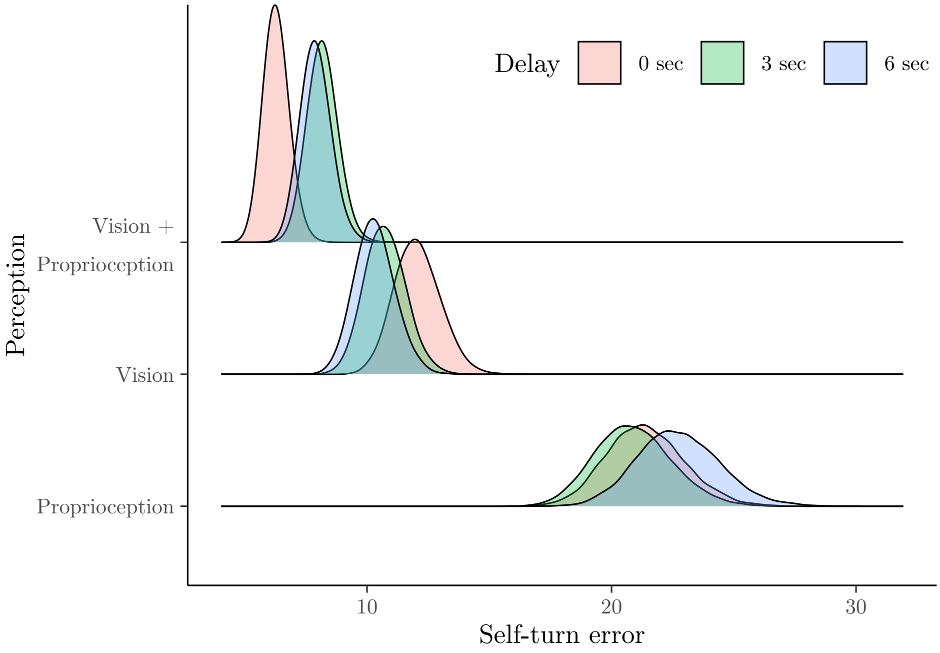


**Figure 12:** Distributions of the predicted means of Self-turn error according to perception and delay ($n_{participants} = 48$; $n_{observations} = 1723$).

**Table 12:** Predicted Self-turn error mean according to perception and delay conditions

| **Conditions** | | **Error** | **95 % BCI** | |
|---|---|---|---|---|
| **Perception** | **Delay** | **Mean** | **Lower** | **Upper** |
| **Proprioception** | 0 sec | 21.39 | 18.45 | 24.66 |
| | 3 sec | 20.89 | 18.03 | 24.16 |
| | 6 sec | 22.75 | 19.61 | 26.24 |
| **Vision** | 0 sec | 12.06 | 10.34 | 13.97 |
| | 3 sec | 10.74 | 9.22 | 12.45 |
| | 6 sec | 10.31 | 8.81 | 11.98 |
| **Vision + Proprioception** | 0 sec | 6.28 | 5.39 | 7.30 |
| | 3 sec | 8.17 | 7.05 | 9.39 |
| | 6 sec | 7.90 | 6.81 | 9.15 |

*Note:* $n_{participants} = 48; n_{observations} = 1723$

To evaluate if there is a plausible difference in the different conditions, Bayesian pairwise comparisons are reported in Table 13. Only in Vision+Proprioception, participants are expected to make less Self-turn errors in the "0 sec" delay condition compared to the "3 sec" and "6 sec" delay conditions. In all the other conditions, the differences are less plausible as the range of most plausible values includes zero values.

**Table 13:** Comparisons between perception and delay conditions

| **Comparisons** | | | **95 % BCI** | |
|---|---|---|---|---|
| **Perception** | **Delay** | **Mean** | **Lower** | **Upper** |
| **Proprioception** | 6 sec - 0 sec | 1.36 | -2.26 | 4.93 |
| | 6 sec - 3 sec | 1.86 | -1.67 | 5.45 |
| | 3 sec - 0 sec | -0.50 | -3.95 | 2.94 |
| **Vision** | 6 sec - 0 sec | -1.75 | -3.64 | 0.10 |
| | 6 sec - 3 sec | -0.43 | -2.20 | 1.30 |
| | 3 sec - 0 sec | -1.31 | -3.21 | 0.56 |
| **Vision + Proprioception** | 6 sec - 0 sec | 1.62 | 0.39 | 2.91 |
| | 6 sec - 3 sec | -0.27 | -1.68 | 1.12 |
| | 3 sec - 0 sec | 1.89 | 0.64 | 3.19 |

*Note:* $n_{participants} = 48; n_{observations} = 1723$

To quantify the relative difference between conditions, the ratios of the Self-turn errors are reported in Table 11.

**Table 14:** Ratio of the Self-turn errors between the different conditions

| **Condition** | | **Ratio** | **95 % BCI** | |
|---|---|---|---|---|
| **Perception** | **Delay** | **Mean** | **Lower** | **Upper** |
| **Proprioception** | 6/0 sec | 1.07 | 0.90 | 1.25 |
| | 6/3 sec | 1.09 | 0.93 | 1.28 |
| | 3/0 sec | 0.98 | 0.83 | 1.15 |
| **Vision** | 6/0 sec | 0.86 | 0.73 | 1.01 |
| | 6/3 sec | 0.96 | 0.81 | 1.13 |
| | 3/0 sec | 0.89 | 0.76 | 1.05 |
| **Vision + Proprioception** | 6/0 sec | 1.26 | 1.06 | 1.49 |
| | 6/3 sec | 0.97 | 0.81 | 1.15 |
| | 3/0 sec | 1.31 | 1.09 | 1.55 |

*Note:* $n_{participants} = 48; n_{observations} = 1723$

## Session Information

```r
sessionInfo(package = NULL)
```

```
## R version 4.0.2 (2020-06-22)
## Platform: x86_64-apple-darwin19.5.0 (64-bit)
## Running under: macOS Catalina 10.15.6
##
## Matrix products: default
## BLAS/LAPACK: /usr/local/Cellar/openblas/0.3.10_2/lib/libopenblasp-r0.3.10.dylib
##
## locale:
## [1] en_US.UTF-8/en_US.UTF-8/en_US.UTF-8/C/en_US.UTF-8/en_US.UTF-8
##
## attached base packages:
## [1] grid      stats     graphics  grDevices utils     datasets  methods
## [8] base
##
## other attached packages:
##  [1] rstan_2.21.2         StanHeaders_2.21.0-6 brms_2.14.4
##  [4] Rcpp_1.0.5           latex2exp_0.4.0      gridExtra_2.3
##  [7] gtable_0.3.0         kableExtra_1.3.1     forcats_0.5.0
## [10] stringr_1.4.0        dplyr_1.0.2          purrr_0.3.4
## [13] readr_1.4.0          tidyr_1.1.2          tibble_3.0.4
## [16] ggplot2_3.3.2        tidyverse_1.3.0      knitr_1.30
##
## loaded via a namespace (and not attached):
##   [1] minqa_1.2.4          colorspace_2.0-0     ellipsis_0.3.1
##   [4] ggridges_0.5.2       rsconnect_0.8.16     markdown_1.1
##   [7] base64enc_0.1-3      fs_1.5.0             rstudioapi_0.13
##  [10] DT_0.16              fansi_0.4.1          mvtnorm_1.1-1
##  [13] lubridate_1.7.9.2    xml2_1.3.2           codetools_0.2-18
##  [16] bridgesampling_1.0-0 splines_4.0.2        shinythemes_1.1.2
##  [19] bayesplot_1.7.2      projpred_2.0.2       jsonlite_1.7.1
##  [22] nloptr_1.2.2.2       broom_0.7.2          dbplyr_2.0.0
##  [25] shiny_1.5.0          compiler_4.0.2       httr_1.4.2
##  [28] backports_1.2.0      assertthat_0.2.1     Matrix_1.2-18
##  [31] fastmap_1.0.1        cli_2.2.0            later_1.1.0.1
##  [34] prettyunits_1.1.1    htmltools_0.5.0      tools_4.0.2
##  [37] igraph_1.2.6         coda_0.19-4          glue_1.4.2
##  [40] reshape2_1.4.4       V8_3.4.0             tikzDevice_0.12.3.1
##  [43] cellranger_1.1.0     vctrs_0.3.5          filehash_2.4-2
##  [46] nlme_3.1-150         crosstalk_1.1.0.1    xfun_0.19
##  [49] ps_1.4.0             lme4_1.1-25          rvest_0.3.6
##  [52] mime_0.9             miniUI_0.1.1.1       lifecycle_0.2.0
##  [55] gtools_3.8.2         statmod_1.4.35       MASS_7.3-53
##  [58] zoo_1.8-8            scales_1.1.1         colourpicker_1.1.0
##  [61] hms_0.5.3            promises_1.1.1       Brobdingnag_1.2-6
##  [64] parallel_4.0.2       inline_0.3.17        shinystan_2.5.0
##  [67] curl_4.3             gamm4_0.2-6          loo_2.3.1
##  [70] stringi_1.5.3        dygraphs_1.1.1.6     pkgbuild_1.1.0
##  [73] boot_1.3-25          moments_0.14         rlang_0.4.9
##  [76] pkgconfig_2.0.3      matrixStats_0.57.0   evaluate_0.14
```

```
##  [79] lattice_0.20-41      rstantools_2.1.1     htmlwidgets_1.5.2
##  [82] processx_3.4.4       tidyselect_1.1.0     plyr_1.8.6
##  [85] magrittr_2.0.1       R6_2.5.0             generics_0.1.0
##  [88] DBI_1.1.0            pillar_1.4.7         haven_2.3.1
##  [91] withr_2.3.0          mgcv_1.8-33          xts_0.12.1
##  [94] abind_1.4-5          modelr_0.1.8         crayon_1.3.4
##  [97] utf8_1.1.4           rmarkdown_2.5        readxl_1.3.1
## [100] callr_3.5.1          threejs_0.3.3        reprex_0.3.0
## [103] digest_0.6.27        webshot_0.5.2        xtable_1.8-4
## [106] httpuv_1.5.4         RcppParallel_5.0.2   stats4_4.0.2
## [109] munsell_0.5.0        viridisLite_0.3.0    shinyjs_2.0.0
```

# References


Akaike, H. (1973). Information theory and an extension of the maximum likelihood principle. In *Second International Symposium on Information Theory* (pp. 267–281). Petrov, B. N. and Csaki, F., editors.

Bolker, B., Brooks, M., Clark, C., Geange, S., Poulsen, J., Stevens, M., & White, J. (2009). Generalized linear mixed models: A practical guide for ecology and evolution. *Trends in Ecology & Evolution*, *24*(3), 127–135. https://doi.org/10.1016/j.tree.2008.10.008

Bürkner, P.-C. (2017). Brms: An R package for Bayesian multilevel models using Stan. *Journal of Statistical Software*, *80*(1), 1–28. https://doi.org/10.18637/jss.v080.i01

Etz, A., & Vandekerckhove, J. (2018). Introduction to Bayesian Inference for Psychology. *Psychonomic Bulletin & Review*, *25*(1), 5–34. https://doi.org/10.3758/s13423-017-1262-3

Fong, Y., Rue, H., & Wakefield, J. (2010). Bayesian inference for generalized linear mixed models. *Biostatistics*, *11*(3), 397–412. https://doi.org/10.1093/biostatistics/kxp053

Fox, J. (2016). *Applied regression analysis and generalized linear models* (Third Edition). Los Angeles, SAGE.

Gelman, A., Carlin, J. B., Stern, H. S., Dunson, D. B., Vehtari, A., & Rubin, D. B. (2013). *Bayesian data analysis.* New York, NY, CRC press. https://doi.org/10.1201/b16018

Gelman, A., Goodrich, B., Gabry, J., & Vehtari, A. (2018). R-squared for Bayesian regression models. *The American Statistician*, 1–6. https://doi.org/10.1080/00031305.2018.1549100

Gelman, A., Hwang, J., & Vehtari, A. (2014). Understanding predictive information criteria for Bayesian models. *Statistics and Computing*, *24*(6), 997–1016. https://doi.org/10.1007/s11222-013-9416-2

Gelman, A., & Rubin, D. B. (1992). Inference from iterative simulation using multiple sequences. *Statistical Science*, *7*(4), 457–472.

Hoffman, M. D., & Gelman, A. (2014). The No-U-Turn Sampler: Adaptively Setting Path Lengths in Hamiltonian Monte Carlo. *Journal of Machine Learning Research*, *15*arxiv 1111.4246, 1593–1623.

Kruschke, J. K. (2013). Posterior predictive checks can and should be Bayesian: Comment on Gelman and Shalizi, 'Philosophy and the practice of Bayesian statistics'. *British Journal of Mathematical and Statistical Psychology*, *66*(1), 45–56. https://doi.org/10.1111/j.2044-8317.2012.02063.x

Kruschke, J. K., & Liddell, T. M. (2018). The Bayesian new statistics: Hypothesis testing, estimation, meta-analysis, and power analysis from a Bayesian perspective. *Psychonomic Bulletin & Review*, *25*(1), 178–206. https://doi.org/10.3758/s13423-016-1221-4

Lo, S., & Andrews, S. (2015). To transform or not to transform: Using generalized linear mixed models to analyse reaction time data. *Frontiers in Psychology*, *6*. https://doi.org/10.3389/fpsyg.2015.01171

McElreath, R. (2016). *Statistical Rethinking: A Bayesian Course with Examples in R and Stan* (First). New York, NY, Chapman and Hall/CRC. https://doi.org/10.1201/9781315372495

Neal, R. (2012). MCMC using Hamiltonian dynamics. In S. Brooks, A. Gelman, G. Jones, & X. Meng (Eds.), *Handbook of Markov chain Monte Carlo* (pp. 113–162). New York, NY, Chapman.

Ng, V. K., & Cribbie, R. A. (2017). Using the Gamma Generalized Linear Model for Modeling Continuous, Skewed and Heteroscedastic Outcomes in Psychology. *Current Psychology*, *36*(2), 225–235. https://doi.org/10.1007/s12144-015-9404-0

Piironen, J., & Vehtari, A. (2017). Comparison of Bayesian predictive methods for model selection. *Statistics and Computing*, *27*(3), 711–735. https://doi.org/10.1007/s11222-016-9649-y

R Core Team. (2018). R: A language and environment for statistical computing. *Vienna, Austria.*

Romeijn, J., & van de Schoot, R. (2008). A philosopher's view on Bayesian evaluation of informative hypotheses. In H. Hoijtink, I. Klugkist, & P. Boelen (Eds.), *Bayesian evaluation of informative hypotheses* (pp. 329–357). New York, NY, Springer. https://doi.org/10.1007/978-0-387-09612-4_16

Stan Development Team. (2017a). Stan Modeling Language: User's Guide and Reference Manual.

Stan Development Team. (2017b). Stan: A C++ library for probability and sampling.

van de Schoot, R., Kaplan, D., Denissen, J., Asendorpf, J. B., Neyer, F. J., & van Aken, M. A. (2014). A gentle introduction to Bayesian analysis: Applications to developmental research. *Child Development*, *85*(3), 842–860. https://doi.org/10.1111/cdev.12169

Vandekerckhove, J., Matzke, D., & Wagenmakers, E.-J. (2015). Model Comparison and the Principle of Parsimony. In J. R. Busemeyer, Z. Wang, J. T. Townsend, & A. Eidels (Eds.), *The Oxford Handbook of Computational and Mathematical Psychology*. Oxford University Press. https://doi.org/10.1093/oxfordhb/9780199957996.013.14

Vehtari, A., Gelman, A., & Gabry, J. (2017). Practical Bayesian model evaluation using leave-one-out cross-validation and WAIC. *Statistics and Computing*, *27*(5), 1413–1432. https://doi.org/10.1007/s11222-016-9696-4

Wagenmakers, E.-J., & Farrell, S. (2004). AIC model selection using Akaike weights. *Psychonomic Bulletin & Review*, *11*(1), 192–196. https://doi.org/10.3758/BF03206482

Xu, R. (2003). Measuring explained variation in linear mixed effects models. *Statistics in Medicine*, (22), 3527–3541. https://doi.org/10.1002/sim.1572

Yamashita, T., Yamashita, K., & Kamimura, R. (2007). A Stepwise AIC Method for Variable Selection in Linear Regression. *Communications in Statistics - Theory and Methods*, *36*(13), 2395–2403. https://doi.org/10.1080/03610920701215639
